# The INRAE Centre for Vegetable Germplasm: Geographically and Phenotypically Diverse Collections and Their Use in Genetics and Plant Breeding

**DOI:** 10.3390/plants11030347

**Published:** 2022-01-27

**Authors:** Jérémy Salinier, Véronique Lefebvre, Didier Besombes, Hélène Burck, Mathilde Causse, Marie-Christine Daunay, Catherine Dogimont, Juliette Goussopoulos, Christophe Gros, Brigitte Maisonneuve, Louis McLeod, Fatiha Tobal, Rebecca Stevens

**Affiliations:** Unité de Génétique et Amélioration des Fruits et Légumes, INRAE, 84140 Montfavet, France; Jeremy.Salinier@cirad.fr (J.S.); veronique.lefebvre@inrae.fr (V.L.); didier.besombes@inrae.fr (D.B.); helene.burck@inrae.fr (H.B.); mathilde.causse@inrae.fr (M.C.); Marie-christine.brand-daunay@inrae.fr (M.-C.D.); catherine.dogimont@inrae.fr (C.D.); juliette.goussopoulos@inrae.fr (J.G.); Christophe.gros@inrae.fr (C.G.); brigitte.maisonneuve@inrae.fr (B.M.); louis.mcleod@inrae.fr (L.M.); fatiha.tobal@inrae.fr (F.T.)

**Keywords:** genetic resources, Solanaceae, *Cucumis*, *Lactuca*, diversity, vegetables, genebank

## Abstract

The French National Research Institute for Agriculture, Food and the Environment (INRAE) conserves and distributes five vegetable collections as seeds: the aubergine* (in this article the word aubergine refers to eggplant), pepper, tomato, melon and lettuce collections, together with their wild or cultivated relatives, are conserved in Avignon, France. Accessions from the collections have geographically diverse origins, are generally well-described and fixed for traits of agronomic or scientific interest and have available passport data. In addition to currently conserving over 10,000 accessions (between 900 and 3000 accessions per crop), the centre maintains scientific collections such as core collections and bi- or multi-parental populations, which have also been genotyped with SNP markers. Each collection has its own merits and highlights, which are discussed in this review: the aubergine collection is a rich source of crop wild relatives of *Solanum*; the pepper, melon and lettuce collections have been screened for resistance to plant pathogens, including viruses, fungi, oomycetes and insects; and the tomato collection has been at the heart of genome-wide association studies for fruit quality traits and environmental stress tolerance.

## 1. Introduction

The evolutionary history of vegetable crops is fascinating because it coincides with the birth and development of agriculture and the world history of human migrations and land discovery. For example, the wild ancestor of aubergine (eggplant), *Solanum insanum*, originated in Asia after ancestral species spread from Northeastern Africa two million years ago [1]. Domesticated chili pepper, *Capsicum annuum*, originated in Central–East Mexico more than 6500 years ago [2] and the process of tomato domestication started in Ecuador and Peru with *Solanum pimpinellifolium* before finishing with modern-sized fruit in Mexico [3]. Melon, *Cucumis melo*, was domesticated in both Africa and Asia before arriving in Europe [4,5]. The ancestor of lettuce was domesticated in Southwest Asia, and then some primitive forms were identified in Egypt around 4500 BC. With the Greek and Roman civilisations, lettuce spread rapidly through the Mediterranean, then Western Europe, and became known in America from the late fifteenth century [6,7,8]. Today’s fruit and vegetables bear little resemblance to their undomesticated wild relatives: for example, the *Lactuca* ancestors of lettuce resemble a weed, with spines under the leaves, bitterness and small seeds [9]. The domestication process for the five crops has changed the size of the edible part, total yield and also fruit shape and colour, ripening and plant architecture [10].

The domestication of plants has been at the expense of the plant’s resistance to abiotic and biotic stresses [11] and latterly selection has also changed the organoleptic quality of the product [12]. The conservation of crop wild relatives (CWR) and knowledge of the evolutionary history of species mean that sources of genes for quality, resistance to pests and pathogens or adaptation to environmental conditions can be fully or partially restored by plant breeders, and knowledge at the genomic level facilitates their use. However, the accessibility and exchange of genetic resources is increasingly complicated, being regulated by international laws: exchange or collection of live material can no longer occur spontaneously. This means that ex-situ conservation is necessary, along with clear documentation (including phytosanitary documents, passport data and material transfer agreements) relating to the stored germplasm, particularly since the ITPGRFA (International Treaty on Plant Genetic Resources for Food and Agriculture) and the Nagoya protocol, adopted in 2010 for fair and equitable sharing of genetic resources [13]. These facts explain the importance of vegetable genebanks as a means of conserving and characterising phytogenetic resources.

Vegetable genebanks from around the world, a non-exhaustive list of which is included in Supplementary Table S1, conserve part of the diversity found in species of local and international importance. In France, a network called ‘RARe’ regroups the French Biological Resource Centres for agricultural, environmental and life sciences (https://www.agrobrc-rare.org/ (accessed on 5 December 2021)). The vegetable genebank situated in Avignon in the south east of France, the INRAE Centre for Vegetable Germplasm—or Centre de Ressources Biologiques Légumes in French—conserves seeds for five vegetable crops. Three of these are from the Solanaceae family: aubergine is a staple food for much of Africa and Asia, pepper is an important crop worldwide, used fresh or dried as a spice, and tomato is one of the world’s most produced fruit vegetables and used fresh or transformed in sauces and conserves. Melon (from the Cucurbitaceae family), grown worldwide in a diversity of forms and consumed as a sweet or non-sweet fruit or vegetable, and lettuce (from the Asteraceae family) complete the list of species conserved and are important for nutritional diversification [14]. More generally, vegetables are an essential part of a balanced diet and the collections are witness to progress in the domestication of vegetable crops and their adaptation to the environment. The need for plants to suit new environmental or agricultural conditions is proof of their value.

The aim of this paper is to unite the information on the five collections conserved by the INRAE Centre for Vegetable Germplasm in a single article, which has not been accomplished previously. The collections are at the heart of much of the research carried out within the Genetics and Breeding of Fruit and Vegetables research unit and form the basis of many collaborations with both research institutes and plant breeders. We will include the history of the genebank’s constitution, the characteristics of the material now present, the methods of conservation, the descriptors used, current databases and finally a spotlight on how the material has been used in different research programmes.

## 2. Overview and Origins of the Five Collections

**Figure 1:**
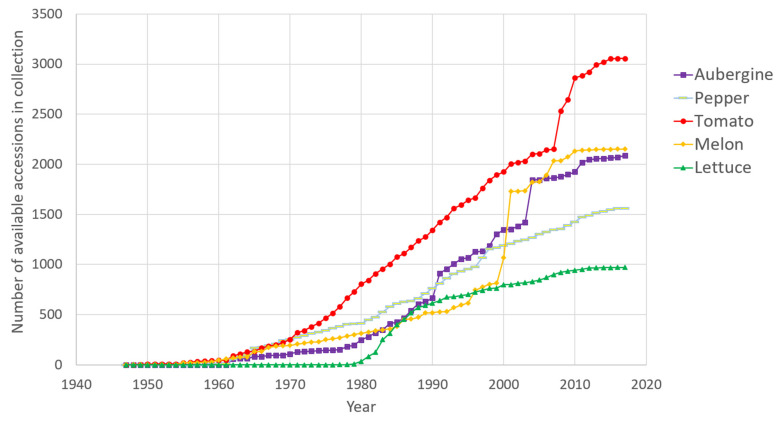
Number of available accessions for each collection by year of introduction: each crop appears with a different colour (see legend). Note that the introduced accessions that have since been ‘lost’ do not appear.

**Figure 2:**
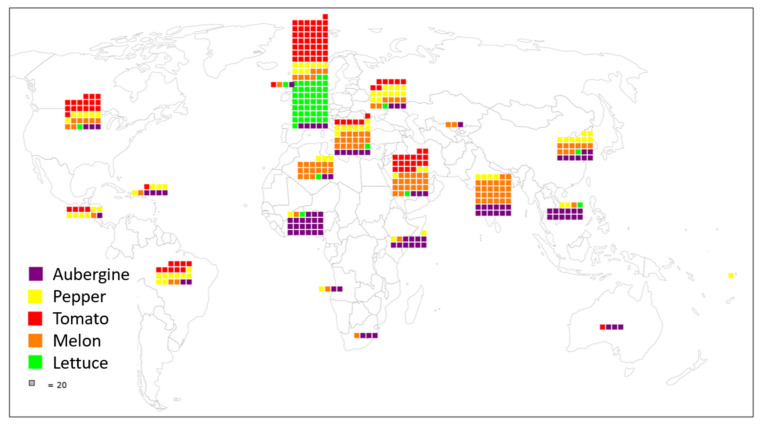
Supposed geographic M49 region of origin or of breeding of the accessions using the colour code shown in the legend. Each square corresponds to 20 accessions. Note that sometimes the recorded origin is that of the collection, even if the accession comes from a foreign country.

Each collection has developed independently over different periods, having been built up by individual researchers working on different research programmes. Generally, the collections started in the 1960s and 1970s and grew rapidly at the end of the last century; introductions have mostly slowed since 2010 (Figure 1). The origins of accessions seen in Figure 2 are closely linked with the domestication/diversification centres for each crop.

Table 1 shows the number of accessions by geographic region of origin based on the M49 regions of the United Nations Statistics Division (standard country or area codes for statistical use) and Supplementary Figure S1 shows the number of accessions by precise country of origin and by continent. There are two points of note on the origin of an accession: firstly, the origin can correspond to the donating genebank’s origin when historical passport data are not available, and, secondly, some accessions resulting from breeding programmes mixing parents of different origins are listed as being of unknown origin, which was seen as preferable to using the local geographic origin: this is the case for both aubergine and lettuce.

**Figure 3:**
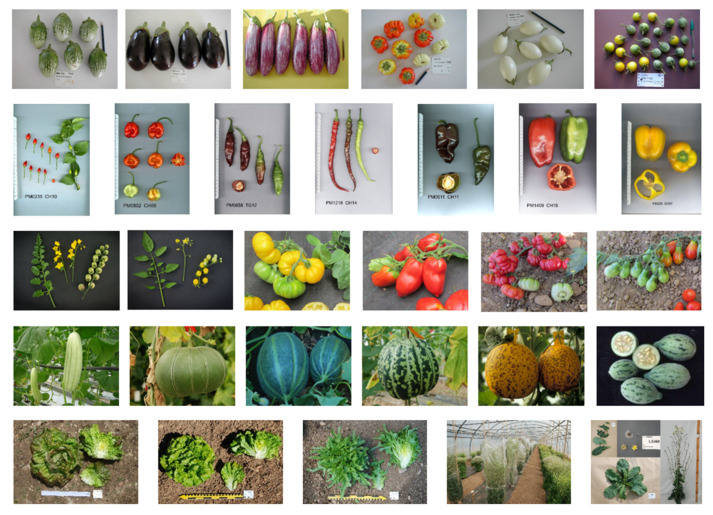
Illustrations of examples of the phenotypic diversity present in the five collections. (**Top**) aubergine collection, from left to right—MM 01560: KOPEK PUTIH/TS58/X452; MM 00133: DE BUKAREST = BUCA; MM 00500: ZEBRINA LONGUE; MM 01737: AUBERGINE LOCAL OP10 (ABIDJAN); MM 00662: BIRM/S.0657; MM 01192: DIEGO ANTSINANARA (MARCHE). (**Second from top**) pepper collection, from left to right—PM0235: CHILI ICONO; PM0802: SAFI; PM0658: BOTIJILLO TINTO; PM1216: EX HD(YWx702)11; PM0611: MULATO ROQUE; PM1409: VANIA; PM0225: PEPERONE DI LUCCA. (**Middle line**) tomato collection, from left to right—T300077: LA1274 *S. peruvianum*; T300165: LA1447: *S. cheesmanii*; T101431: Marmande Jaune; T101560: Poivron des Andes; T102434: EA2679; T102446: EA2751. (**Second from bottom**) melon collection, from left to right—Unknown; ME0857: MR-1; ME2343: ACEM; ME2369: DAMIAN GUA; ME1038: SVI 0024; wild African accession. (**Bottom**) lettuce collection, from left to right—LC0251: butterhead cv Grosse brune têtue; LC0152: Batavia, cv craquante d’Ecully; LC0206: oak leaf, cv Feuille de chêne espagnole; Production of seed in insect-proof tunnel; LC0858: *L. virosa*, LS360.

The collections are rich in phenotypic diversity: many lines have been fixed for morphological characteristics often related to fruit size, shape or colour. Some of the visual diversity of a selection of our collections is shown in Figure 3 and includes wild or other cultivated species. The morphological diversity can be used to classify the collection into groups based on morphological data such as fruit colour and shape (Supplementary Figure S2), which is useful in certain cases—for example, for breeding or for comparing with genotyping data.

Table 2 summarises the number of accessions for the main individual species and the number of crop wild relatives, and Supplementary Figure S3 provides extra information on the number of accessions present in the collections for individual species.

## 3. Summary of the Individual Collections

Each collection is described in detail below with information on geographic diversity, taxonomy and scientific resources available: core collections are available for aubergine, pepper and tomato [15,16]. Many mapping progenies have been created for research purposes and are available for collaborative projects.

(i). **The aubergine collection** (**2388 accessions**) contains two introductory peaks: one from 1991, which corresponds to the scientific collaboration with the taxonomist Richard N. Lester of the University of Birmingham, UK, and the second in 2004, when the collection of *Solanum* species related to aubergine were transferred from the University of Birmingham to INRAE [17]. More recently, we have introduced new aubergine accessions from the H2020 G2P-SOL project (http://www.g2p-sol.eu/ (accessed on 5 December 2021)). Most of the aubergine accessions originate from Africa and Asia. The aubergine collection is notable amongst the five collections because it includes a large proportion of crop wild relatives (26%). This number of CWR is rare in other germplasm centres, mostly because of the difficulties in maintaining them. The collection includes more than 1000 accessions principally from Africa (related cultivated species including *Solanum macrocarpon* L., *Solanum aethiopicum* L., *Solanum scabrum Mill.).* These species are indigenous African leafy vegetables and/or fruits: *S. aethiopicum* and *S. macrocarpon* provide a usable secondary gene pool for the improvement of *S. melongena.* Approximately 500 accessions, representing over a hundred wild species, the majority related to cultivated aubergine, complete the collection, as well as accessions of other Solanaceae of interest (*Atropa*, *Datura*, *Lycium*, *Nicandra*, *Physalis* and *Withania*) [18]. A word of caution must be added about the taxonomy of *Solanum* species. The taxonomic classification of aubergine dates back to the work of RN Lester in the 1990s; the nomenclature has evolved since the early 2000s and the taxonomic status of several taxa is unclear [19,20].

The aubergine scientific collection contains five double-haploid populations and F1, F2 and backcrosses of six biparental progenies. A total of 706 aubergine accessions from our collection were supplied to the G2P-SOL project, of which 106 are included in the final core collection from the project. The project has and will supply low- and high-density genotyping data, which extends the knowledge available on the whole collection.

(ii). **The pepper collection** (**2188 accessions**) is representative of domestication centres (South and Central America). In pepper, the collection focuses on *Capsicum annuum* (76% of accessions), with a large collection, rich in phenotypic and geographic variability, that is easily exploited in breeding programmes. Eleven species of *Capsicum* are available in the collection, including the five cultivated species (C. *annuum*, *C. frutescens*, *C. chinense*, *C. baccatum* and C. *pubescens*) [21]. Recombinant inbred lines in pepper [22] have allowed the evaluation of fruit traits [23] and resistance to *Phytophthora* species [24,25]. Similarly to aubergine, 912 INRAE accessions (889 cultivated) were included in the G2P-SOL project, of which 59 are included in the final core collection [26]. The genotypic diversity in pepper from the G2P-SOL core collection maximises the diversity of around 10,000 accessions from 10 genebanks and research institutes from around the world in a collection of 423 mostly *C. annuum* accessions [26]. INRAE is the official distributor of the G2P-SOL pepper core collection. Another core collection of over 280 accessions has been constructed with INRAE material [27].
(iii). **The tomato collection** (**3410 accessions**) is representative of its domestication centre (South and Central America). In tomato, the number of wild-relative species is lower than in the other collections but a good diversity of *S. pimpinellifolium* is available. For tomato, *S. peruvianum* has been separated into four species, including two new species, *S. arcanum* and *S. huaylasense*, which requires database information to be corrected: taxonomic identification is therefore an ongoing process [28]. More than 500 accessions have been genotyped with the SolCap Illumina array and a core collection of 160 accessions constructed and amply characterised (see below) [29]. The tomato scientific resources include progenies of recombinant inbred lines [30], advanced backcrosses, intra- and interspecific progenies and multi-parent progenies (MAGIC) [31] representing more than 1000 accessions.
(iv). **The melon collection** (**2359 accessions**) comes from all around the world, particularly Africa and Asia, and includes around 100 genotypes of wild *C. melo* (mostly from the agrestis cultigroup), which are compatible for crossing with cultivated melons. Recombinant inbred lines obtained by crossing distant melon lines have been created and studied for many segregating agronomic traits and for monogenic as well as quantitative pest and disease resistance [32,33,34,35,36,37]. A mutant melon collection obtained by chemical EMS mutagenesis of an INRAE Charentais melon line includes more than 7000 M2 families and is useful for the functional validation of genes or for generating new diversity [38,39,40,41].
(v). **The lettuce collection** (**948 accessions**) comes essentially from Europe. For cultivated lettuce, the introduction year is unknown for 151 cultivars received before 1980; for wild *Lactuca* species, 64% of the wild accessions (mainly of *L. serriola*) were collected directly by INRAE, mainly in France in the 1980s. Out of the 479 introduced wild accessions, 343 collected accessions and 136 received from other laboratories, only 248 are still present with seed stock in the 2020s: seed for many accessions arriving in the 1970s was lost because the storage at room temperature before 1984 was inadequate for long-term conservation in *Lactuca*. In lettuce, the 704 cultivated accessions are *L. sativa*, with many modern cultivars cultivated in Western Europe over the last 40 years. For wild lettuce, the precise collection site is known for 92% of the INRAE accessions. There are 11 *Lactuca* species, including the three species mainly used by breeders: *L. serriola*, *L. saligna* and *L. virosa*. The lettuce collection is completed by 15 accessions from other genera of the Asteraceae family (*Chondrilla*, *Mycelis*, *Sonchus*). The lettuce collection contains a few lines with resistance to potyviruses or *Bremia lactuca* identified in *L. virosa* and introgressed into a cultivated background [42,43,44].

## 4. Collection Management

**Figure 4:**
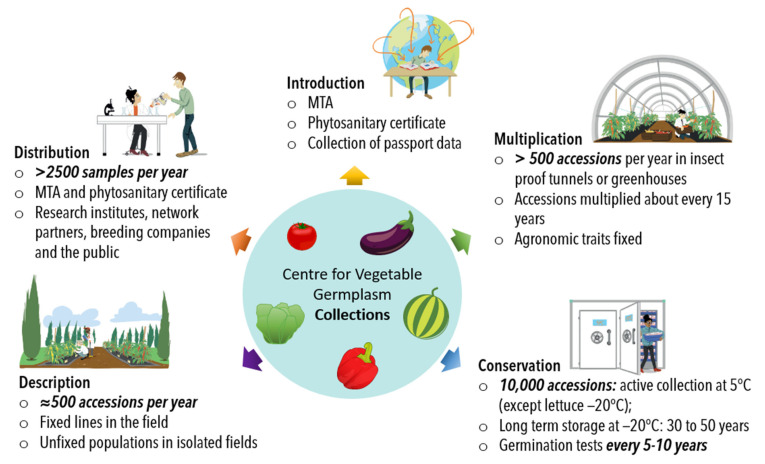
Current procedures for management of the collections at the INRAE Vegetable Germplasm Centre, including the major missions of introduction, multiplication, description, conservation and distribution and with an estimation of the number of samples or accessions involved. The multiplications are mostly carried out by self-pollination. Illustrations Camille Ulrich—Copyright INRAE.

The genebank follows typical procedures for seed storage, multiplication and distribution [45]. An overview of the processes and procedures is provided in Figure 4, with an indication given regarding the number of accessions or samples concerned by each process for a typical year. These figures are specific to the INRAE Centre for Vegetable Germplasm.

(i). Conservation of seed stocks

The genebank focuses on medium- and long-term storage (4 °C and 50% relative humidity for ~15 years or −20 °C and 50% relative humidity for >30 years [46]) with procedures for checking germination rates following long-term storage (after one year and then every 5 or 10 years depending on the results). Safety duplicates are held at a genebank centre based in Montpellier, France.

(ii). Regeneration of seed stocks

Regeneration by seed multiplication is performed mostly by controlled self-pollination in insect-proof greenhouses (three plants per accession, two for lettuce). For accessions that are regenerated as wild populations, i.e., those accessions that were harvested from a mixed genepool that potentially contains many rare alleles and/or accessions that are self-incompatible, we use isolation fields with natural pollinators. We aim for a minimum of 600 seeds per accession. Twenty seeds are used in germination tests.

The management of a collection of wild relatives of *Solanum* is complex: problems arise at numerous levels—for example, difficulty in obtaining accessions, misidentification, poor germination, physiological problems with flowering, pollination and fruit set [47]. Many wild accessions missing from the collection are endangered because of the eradication of their natural habitat, notably in East Africa [48]. Many species cannot be regenerated in the short culture periods available in the greenhouses or fields in Avignon; this is particularly true for the majority of *Solanum* species native to Australia and many species of tropical origin. New culture conditions for those species that are difficult to regenerate have been investigated in a recent project (https://www6.paca.inrae.fr/gafl_eng/Partnerships-and-Projects/Closed-Projects/SESAM (accessed on 5 December 2021)) in which we have varied the photoperiod, growing season or watering regime to obtain success in saving some accessions.

(iii). Descriptions

Collections are systematically characterised using botanic and primary descriptors (fruit, flower, plant and leaf characteristics). A large percentage of the collections has been described using descriptors from either the International Plant Genetic Resources Institute, the International Union for the Protection of New Varieties in Plants or with in-house descriptors. The supplementary dataset includes the list of descriptors used for each collection (Supplementary Figure S4) and the proportion of accessions that have been described. Fruit diversity and colour are a particular focus for descriptions [23,26,49,50,51] and we are now establishing techniques for phenotyping root system architecture [52]. Specific ways of describing wild lettuce and aubergine, which have characteristics quite different to the cultivated species, have also been put into place (Supplementary Figure S4). Cultivated lettuce has been described for bolting and flowering characteristics; the heading characters were scored on progeny, either in the field for cultivars bred for outdoor culture, or under plastic tunnels for cultivars bred for protected cultures (Supplementary Figure S4). For the five collections, secondary descriptors include criteria such as pest and pathogen resistance (Supplementary Figure S4). Fixing of traits of agronomic interest (for example, fruit colour or shape) has been carried out for most of the accessions when possible, in order to complete phenotypic gaps in the collections.

(iv). Networks and sub-collections

Each INRAE collection is wholly or partially regenerated and/or described in partnership with private breeding companies, the French organisation ‘Variety and Seed Study and Control Group’ (GEVES) and the CIRAD. With the INRAE Centre for Vegetable Germplasm, a different subset of these partners forms three national networks: (i) the Fruit Solanaceae network (created in 1996), (ii) the melon network (since 1997) and (iii) the *Lactuca* network (created in 2020). Each network partner not only contributes to the network by multiplying seeds and describing accessions each year but also shares extra varieties or accessions with the network’s members. The French national collection (approximately 100 accessions of patrimonial value) is defined by the network members and includes accessions from INRAE and the network’s collections. The national collection is freely available to all parties and the public and appears in the databases (see below). In addition, the aubergine national collection has been submitted to the International Treaty on Plant Genetic Resources for Food and Agriculture (ITPGRFA), to encourage the open exchange of plant genetic resources for food use.

(v). Seed and data sharing

Online local databases are used for storing passport data, descriptions and information on seeds (seed batch date, seed quantity, exchange, storage conditions and germination rate). Our local databases are linked to national or European databases where part of the collections are found (for the moment often only the national collection). These databases contain accession names and passport data as a minimum and currently include Siregal (https://urgi.versailles.inra.fr/siregal/siregal/grc.do (accessed on 5 December 2021)), where phenotyping data are also available, Florilège (http://florilege.arcad-project.org/fr (accessed on 5 December 2021)), ECPGR (https://www.ecpgr.cgiar.org/ (accessed on 5 December 2021)) and Eurisco (https://eurisco.ipk-gatersleben.de/ (accessed on 5 December 2021)). As many changes are occurring in the management of these databases and the links between them, we have provided a list of our accessions and passport data in Supplementary Table S2. This includes the national, network and INRAE collections. Please contact us for the distribution conditions specific to each accession.

Seeds can be ordered via our local website: https://www6.paca.inrae.fr/gafl_eng/Vegetable-Germplasm-Centre/Use-our-genetic-resources (accessed on 5 December 2021). A charge is made for sending accessions, which are provided with a phytosanitary passport or certificate and MTA as necessary.

We estimate that approximately 2000–3000 samples are sent annually to partners outside the research unit, of which around 50% are for private research companies, nearly 20% are for international and national research laboratories and around 5% go to members of the general public, associations and amateurs. The remaining 25% of samples are supplied to other INRAE laboratories.

## 5. The Collections as Material for Scientific Study

The genetic resources are used by INRAE, research institutes, universities and breeding companies. The collections are currently used or studied for the following themes.

(i). Domestication and structure of the collections

Solanaceae genetic resources conserved at INRAE have been used to investigate domestication scenarios by analysing transcriptome data from aubergine, pepper and tomato with their close wild relatives to show both common and species-specific demographic changes, the latter being particularly true for aubergine [53]. Geographic isolation has played a role in determining the genetic structure of aubergine populations; there is also evidence for outcrossing in aubergine between wild and cultivated gene pools [20,54,55,56].

In pepper, 1352 accessions of our collection, representing 11 *Capsicum* species with wide geographic diversity, were genotyped using 28 microsatellite markers. This collection was assigned to six clusters, with three clusters for *C. annuum* and three additional clusters separating the other main species, including the cultivated species and wild relatives, according to their taxonomic classification (*C. frutescens*/*C. chinense*, *C. baccatum*, *C. pubescens*). The three *C. annuum* clusters were significantly distinct for plant and fruit descriptors corresponding to cultivar types, showing that the genetic structure of cultivated pepper has been affected by human selection in primary as well as secondary diversification centres [21]. A total of 869 accessions from our collection, together with more than 9000 accessions from nine other collections worldwide, were genotyped with more than 26,000 GBS-derived SNPs in the G2P-SOL project. This study revealed a reticulate interbreeding history in *C. annuum* and a differentiation between two major genepools (i.e., European and Central American/Asian). It also demonstrated that approximately 80% of the accessions we maintain at INRAE are not represented in the main world collections [26].

In tomato, where the phenotypic effects of domestication have been well documented [57,58], it has been shown that domestication has affected gene expression within gene regulatory networks: changes in gene co-expression levels were associated with lower nucleotide diversity, often because of the fixation of useful mutations during domestication [53,59]. Tomato also appears to have been domesticated in a two-step process: first in South America and then in Mesoamerica [60]. A similar reduction in nucleotide diversity has been seen in candidate genes involved in meristem development in cultivated compared to wild tomato: around half the genes analysed revealed footprints of selection and polymorphisms putatively involved in fruit size variation by showing negative Tajima’s D and a reduction in nucleotide diversity in cultivated tomato [61,62].

Genotyping of 713 melon accessions has given information on the collection’s genetic subdivisions and led to the hypothesis that melon has probably been domesticated at least twice [63]. More recently, one hundred accessions of our collection, together with more than 1000 accessions worldwide, have been re-sequenced: the data confirmed the two domestications in Africa and Asia and suggested the occurrence of a third domestication event in India [5]. Iconographical and textual sources suggest the presence of melon in the Mediterranean basin dating back to antiquity and are illustrated by two melon types in Italy in the late medieval times: melons of the Chate group, the likely ancestor of our sweet melons, and elongated and unsweet snake melons, still appreciated in East and North Africa [64]. Complementary studies have shown the genetic diversity available within melon cultigroups [65,66] and the phenotypic diversity, which is greater in cultivated melon than wild melon [51].

(ii). Resistance to plant pests and pathogens

In aubergine, the collections are of particular interest in screening for *Ralstonia solanacearum* resistance and mapping populations have been created to this end: *R. solanacearum* (bacterial wilt, southern wilt or potato brown rot) causes major yield losses in the tropics and subtropics but is also a threat in temperate climates [67,68,69]. Numerous strains or phylotypes exist, and, whereas one aubergine variety may be resistant or partially resistant to one strain, it is often not resistant to all strains. The aubergine collection is a source of resistance genes against *Ralstonia*: from a subset of 10 genotypes, none controlled all strains but some are able to control the most virulent strains (six accessions were totally resistant to six strains) [70,71,72,73]. Crosses between resistant and susceptible parents have generated recombinant inbred lines [72] for QTL mapping related to resistance in different environments; a major resistance gene, *ERs1*, has been identified. Further sequencing and QTL mapping for resistance have shown that both broad-spectrum and strain-specific QTLs exist and that, by combining three or more QTLs, large-spectrum resistance could be obtained [74,75]. Remarkably, aubergine accession Ceylan SM 164 was resistant to all six strains. Aubergine accessions SM6, Surya and AG91-25, as well as pepper accessions CA8 and MC4, were moderately to highly resistant to all six strains [76].

The pepper germplasm has been shown to include a diversity of genetic backgrounds favourable to resistance durability to pests and pathogens. In the case of potato virus Y, the evaluation of a core collection of *Capsicum annuum* landraces showed breakdown frequencies of given resistance alleles that varied from 0 to 53% [15]. In the same core collection, genome-wide association studies detected SNPs associated with the presence of the virus at inoculation and/or systemically [27]. Screening of 1179 accessions of the pepper collection for quantitative resistance to *P. capsici* revealed 26 new sources of resistance [77] and furthermore a key conserved QTL cluster has been shown to exhibit broad-spectrum resistance to *P. capsici* [78]. In a similar way, double-haploid lines obtained from the F1 hybrid of a resistant accession (H3) crossed with a susceptible accession (Vania) revealed QTLs for resistance to powdery mildew due to *Leveillula taurica* [79]. Other double-haploid progenies have been used to study the genetics of resistance to viruses such as potato virus Y, tobacco mosaic virus, tomato spotted wilt virus, cucumber mosaic virus and pepper veinal mottle virus [80,81,82,83,84], and *P. capsici* [85]. Within the G2P-SOL project, the final core collection is to be phenotyped for agronomic traits and biotic stress resistance, including to *Phytophthora capsici*, *Leveillula taurica*, cucumber mosaic virus and *Meloidogyne incognita* by INRAE and *Verticilium dahliae*, pepper mild mottle virus, *Fusarium oxysporum* and tomato spotted wilt virus by other partners.

The melon collection has been extensively evaluated for resistance to pests and pathogens, including several viruses (cucumber mosaic virus; potyviruses such as the zucchini yellow mosaic virus; and the whitefly-transmitted begomoviruses, melon chlorotic mosaic virus, tomato leaf curl New Delhi virus and watermelon chlorotic stunt virus); fungi such as Fusarium wilt and downy and powdery mildew, and also insects [86,87,88,89,90,91]. QTLs have been detected for both whitefly and aphid resistance in a biparental population: a major QTL affecting aphid behaviour and biotic potential co-localises with the *Vat* gene, although resistance is polygenic [32,92,93,94,95]. In melon, an example of a success story is the cloning of the *Vat* gene, which was shown to be a CC-NBS-LRR gene mediating both resistance to aphid infestation and virus infection using *Aphis gossypii* as a vector [94,96].

The lettuce collection in particular has been a target for screening for resistance to plant diseases including viruses, oomycetes, bacteria, nematodes and fungi (summarised in Table 3) [42,97,98,99,100]. Breeding for resistance in lettuce is a priority and many resistance gene sources have been reported, particularly in wild lettuce species, for potyvirus, *B. lactucae* and *Meloidogyne incognita* [100,101,102]. When resistance is found in wild species, the compatibility with *L. sativa* determines the success of breeding programmes: several species are compatible with *L. sativa* (*L. serriola*, *L. saligna* and *L. virosa*) and can therefore be used, but the crosses are only straightforward with *L. serriola*. The hybrids with *L. virosa* are sterile and often show physiological disorders such as necrosis or stunted plants, which are difficult to eliminate in any progenies that are obtained [101]. Resistant genes from *L. virosa* have been introgressed into a butterhead background to produce lines with new resistance to *Bremia* [103] and two potyviruses [43,44]. More than 400 lettuce accessions have also been tested with three stimulators of plant defences; some interesting protection against *B. lactucae* was obtained with a few cultivars [104].

(iii). Floral biology and crossing compatibility

The outcrossing potential of aubergine was investigated using 23 populations of wild weedy aubergine [54]. Controlled crosses with cultivated aubergine resulted in seed set and viable F1 progeny. The exerted stigmas of wild aubergines are likely to promote outcrossing under natural conditions, highlighting the risk of growing Bt transgenic plants in Southern India, where wild and cultivated aubergines coexist.

Variability in the melon germplasm collection (497 accessions of *C. melo*) has been used to validate a locus controlling sex determination in melon: within C. melo, most plants are monoecious (single sex flowers) or andromonoecious (male and hermaphrodite flowers). This trait is under the control of a recessive locus and a single-nucleotide polymorphism in the CmACS7 gene, which co-segregates with the sex determination phenotype of the flowers [53]. The insertion of a transposon, which epigenetically controls the expression of the CmWIP1 transcription factor, was shown to co-segregate with the gynoecious (female flowers only) phenotype [41]. Together with advances in the understanding of sex determination in plants, these studies have provided markers of interest for breeders, facilitating F1 seed production in melon.

The crossing potential of seven accessions of *L. virosa* with 10 lettuce cultivars was compared by using in vitro cultures of immature embryos or harvesting mature seeds. Few F1 progenies were obtained but, after backcrossing to lettuce and self-pollination, some lines with virus resistance were obtained [106].

(iv). Fruit quality and abiotic stress tolerance

In pepper, the collections have been used to show that the gene encoding Capsanthin Capsorubin Synthase is responsible for the yellow colour in pepper [50]. Following work on sensory traits in an intraspecific RIL population [49], several core collections of tomato have been used to evaluate the genetics of fruit quality traits [16,107,108,109,110]. An example is the genetics of fruit metabolite content—including sugars, vitamin C, amino acids and volatiles. These traits, using two different genotyped core collections, one composed mostly of cherry tomato accessions and the other including breeding material, have been shown to be heritable and under the control of multiple QTLs. The studies also pinpointed candidate genes for traits such as fruit malate content and phenylpropanoid volatile production [16,108]. The GWAS studies have paved the way towards predictive genomic selection, which can be used on crops such as tomato: this could be particularly useful for polygenic traits such as fruit quality [111]. The tomato scientific collections have also been evaluated for potential adaptation to environmental stress—in particular, under conditions of limited water [107,112], elevated temperature [113] and, for the MAGIC population, multiple stress conditions [114], revealing candidate genes involved in stress responses.

(v). Selection and breeding

The creation of new vegetable varieties has mostly stopped at INRAE and is now the domain of private plant breeders. However, some of our past creations are well known. Crosses of certain aubergine introductions combined with INRAE breeding in traditional populations allowed the creation of the first French F1 hybrids, registered in the official catalogue in 1973: F1 Bonica with purple globular fruits and F1 Baluroi with medium-length purple fruits. In aubergine, crop wild relatives offer numerous possibilities for the improvement of *S. melongena* [48], including providing resistance to pests and pathogens: the challenge is creating interspecific hybrids (sexual or somatic) and research has been dedicated to this area over recent decades [115,116,117,118].

Crop wild relatives are also particularly good as rootstocks [119]. The Solanaceae family contains many candidates that can be used as rootstocks for cultivated aubergine and tomato. Part of the collection has been screened to find species having a good grafting affinity with aubergine and a number of candidates identified [120,121].

In pepper, our best-known varieties are Lamuyo F1 hybrid (the first pepper hybrid in the world) and Alby (cumulating five resistance genes) as well as the F1 sweet pepper hybrid Ulysse. In tomato, Montfavet H63-5 is an F1 hybrid widely grown in France, and elsewhere, in the 1970s. Recent tomato varieties include Garance and Jouvance (good colour and flavour, firm, eight disease resistance genes) and Terradou (processing tomato, high soluble solids).

The Margot (1988) melon was the first variety to integrate the *Vat* gene for aphid resistance, and breeders have incorporated the resistance into many varieties, currently including more than 50% of Charentais varieties. Our genetic resources are still used for breeding through our private/public networks and particularly the melon network: only the genetic diversity within the species *C. melo* is usable—there are currently no inter-specific crosses.

For lettuce breeding, resistance to Lettuce mosaic virus (LMV), identified in *L. virosa* PIVT1398 [42], was introduced into butterhead lines by INRAE [101]. The NILs with the *Mo3* locus were also resistant to another potyvirus: lettuce Italian necrotic virus (LINV) [44]. Some NILs with the *Mo3* resistance gene have been made available for research and breeding [43].

## 6. Conclusions and Perspectives

**Figure 5:**
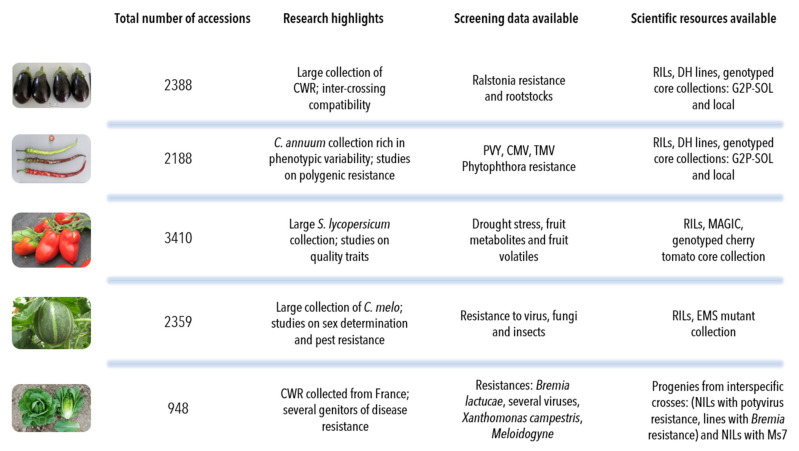
Collection highlights, total number of accessions, screening data and scientific resources available for each of the five collections.

An overview of the highlights of each of the five collections with the scientific data and resources that have been produced is presented in Figure 5. The availability of genotyping data and phenotypic data has enhanced the usefulness and long-term perspectives of the collections, although phenotyping is still to be carried out on the collections: for example, root descriptors are mostly absent and little information is available on the adaptation of the collections to the environment. Projects including phenotyping or genotyping also allow us to improve the collections by eliminating duplicates or by using introductions to cover gaps. We mainly aim at being complementary to other collections in the world, but there is a need to know which accessions are complementary, and this shows the importance of having an inventory of genetic resources (passport data and descriptors) or genotyping data of collections, which also presents several advantages:

- Allows the study of allelic diversity for genes of interest for mining allelic diversity;
- Facilitates the determination of the “uniqueness” of an accession and better identification of duplicates;
- Gives an idea of the phenomenon of introgression between species;
- Can help in the study of core- and pan-genomes.

For the longer-term perspectives for our genetic resource collections at a local level, we can list as priorities:

- Increasing the duration between multiplication cycles by improved conservation;
- Mobile applications for management of collections (seed harvest, descriptions in the field, etc.) and their direct link to databases for improved traceability and quality standards with the aim of obtaining ISO 9001 certification;
- More complete and quantifiable phenotyping by use of image analysis to measure size, shape and colour of plant organs.

We are continuing our efforts to reduce the cost of conservation, guarantee the long-term preservation of crop resources and increase the knowledge on these resources to facilitate their use in research and breeding projects.

## Figures and Tables

**Table 1:** Number of accessions originating from the different regions of the United Nations Statistical Division. Note that sometimes the recorded origin is that of the original collection, even if the accession was introduced from a foreign country. The colour scale ranges from dark (most represented region) to light (least represented region).

Geographic Region	Aubergine	Pepper	Tomato	Melon	Lettuce ^1^
Australia and New Zealand	59		4		
Melanesia		2			
Polynesia	1				
Caribbean	75	79	26	18	
Central America	9	115	97	4	
Northern America	45	102	354	140	5
South America	29	170	208	21	2
Central Asia	4	3		36	5
Eastern Asia	150	151	39	175	9
Southeastern Asia	237	52	4	19	1
Southern Asia	230	69	14	518	
Western Asia	51	57	378	370	18
Eastern Africa	195	50	6	7	
Middle Africa	34	22		12	
Northern Africa	33	85	6	285	3
Southern Africa	60		4	3	
Western Africa	406	41	20	8	1
Eastern Europe	53	222	175	109	1
Northern Europe	3		53	13	5
Southern Europe	106	160	145	303	37
Western Europe	97	165	913	122	224

^1^ Not applicable for modern commercial cultivars (600 cultivated lettuces) because, for a given cultivar, breeding can be carried out in different countries.

**Table 2:** Summary of taxonomy of the collections for the accessions whose taxonomy has been identified.

Collection	Species/Crop Wild Relatives	Number of Species	Number of Accessions
Aubergine	*Solanum melongena*		1211
	*S. aethiopicum*		335
	*S. macrocarpon*		91
	Crop wild relatives—Solanum	109	609
	Other Solanaceae genus (8)	17	24
Pepper	*Capsicum annuum*		1683
	*C. baccatum*		129
	*C. chinense*		159
	*C. frutescens*		86
	*C. pubescens*		28
	Crop wild relatives	6	24
Tomato	*Solanum lycopersicum*		3095
	Crop wild relatives	9	285
Melon	*Cucumis melo* ^1^		2359
Lettuce	*Lactuca sativa*		712
	Crop wild relatives—*Lactuca*	10	225
	Crop wild relatives—other genus	3	15

^1^ includes 91 *Cucumis melo* subsp. agrestis (wild melon).

**Table 3:** Screening of the lettuce collection for resistance to pests and pathogens.

Plant Disease	Number of Accessions	Comment
*Bremia lactucae* Reactive cultivars to stimulator of plant defences (SDP) for *Bremia* protection	400 and 66 accessions within the European Evaluation Network project 402 cultivars tested with 3 SDP	EVA projet (2019–2023) https://www.ecpgr.cgiar.org/european-evaluation-network-eva/eva-networks/lettuce/ (accessed on 5 December 2021) Some reactive cultivars with good protection against *Bremia* [104]
Potyvirus lettuce mosaic virus Potyvirus lettuce Italian necrotic virus	231 (116 cultivated and 115 wild) 20 (11 cultivated and 9 wild)	*L. virosa* PIVT1398 resistant to all lettuce mosaic virus strains [42]. One resistant: PIVT1398 [97] Same *Mo3* locus, introgressed from *L. virosa*, confers resistance to LMV and to LINV [43]
*Xanthomonas campestris*	986 (789 cultivated and 197 wild)	Few genitors in cultivars [99] QTL analysis in RIL population [105]
*Meloidogyne incognita*	569 (409 cultivated and 160 wild)	Resistance found in *L. sativa* and *L. serriola* [100]

## Data Availability

Not applicable.

## Supplementary Materials

The following are available online at https://www.mdpi.com/article/10.3390/plants11030347/s1, Figure S1: Number of accessions and their origins by country; Figure S2: Morphological groups (aubergine—fruit colour; pepper—fruit shape, colour and pungency; tomato—fruit shape and colour; melon—cultigroups; and lettuce—cultigroups); Figure S3: Number of accessions and their taxa or species; Figure S4: Qualitative and quantitative descriptor lists used for each of the five crops and the proportion of accessions that have been described. Table S1: Non-exhaustive list of vegetable genebanks worldwide; Table S2: List of the accessions for each collection with passport data (Excel file).
