# Supplementary material for: The INRAE Centre for Vegetable Germplasm: Geographically and Phenotypically Diverse Collections and Their Use in Genetics and Plant Breeding
Source: Plants (Basel). 2022 Jan 27;11(3):347. doi: 10.3390/plants11030347 (PMC8838894; doi:10.3390/plants11030347)
Supplement: Supplementary file 1 [file plants-11-00347-s001.zip › suppFiguresResubmission.pptx]

## Slide 1
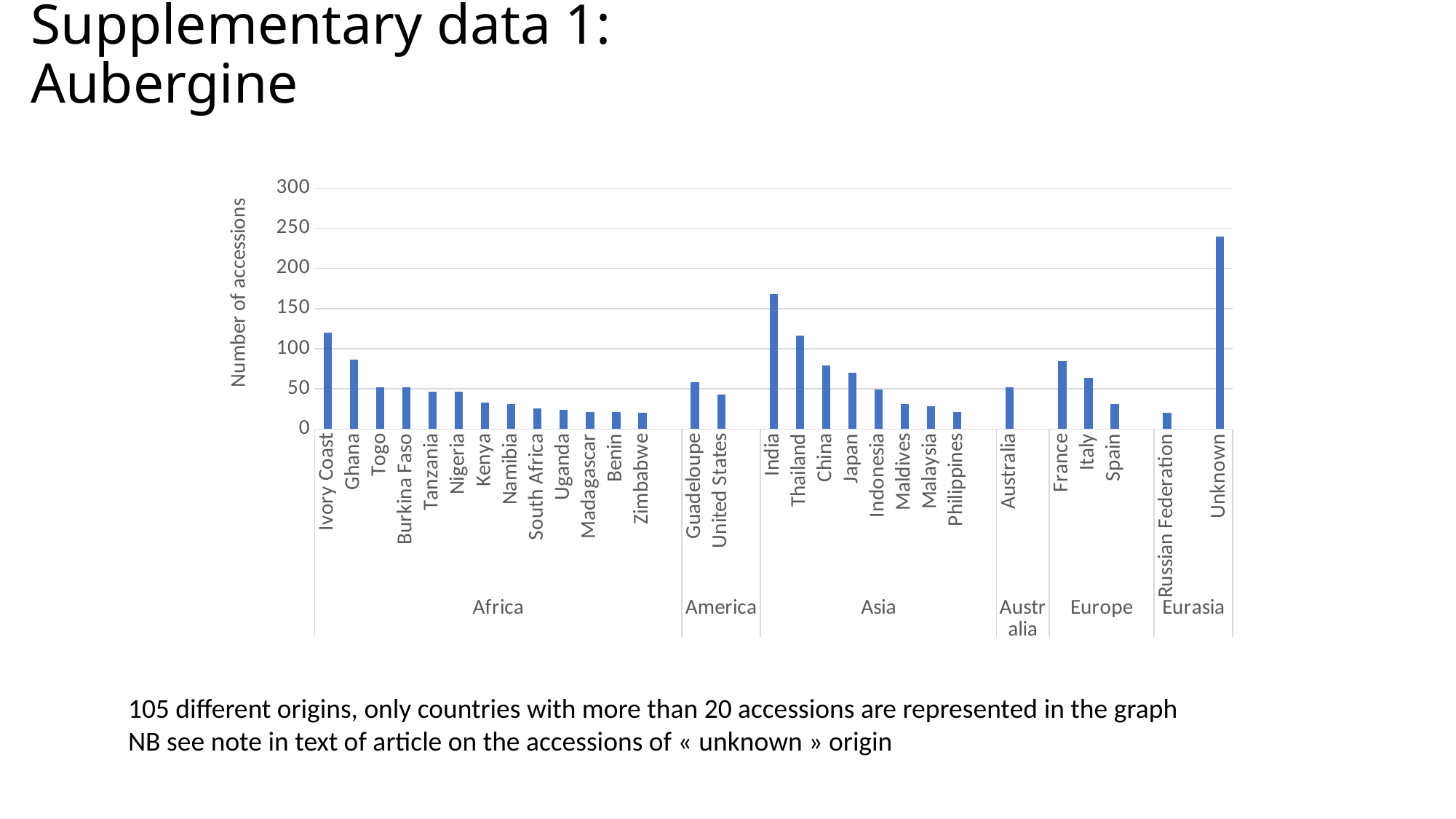

# Supplementary data 1: Aubergine
### Chart
| Category | |
|---|---|
| Ivory Coast | 120.0 |
| Ghana | 86.0 |
| Togo | 52.0 |
| Burkina Faso | 52.0 |
| Tanzania | 47.0 |
| Nigeria | 47.0 |
| Kenya | 33.0 |
| Namibia | 31.0 |
| South Africa | 26.0 |
| Uganda | 24.0 |
| Madagascar | 21.0 |
| Benin | 21.0 |
| Zimbabwe | 20.0 |
| | None |
| Guadeloupe | 58.0 |
| United States | 43.0 |
| | None |
| India | 168.0 |
| Thailand | 116.0 |
| China | 79.0 |
| Japan | 70.0 |
| Indonesia | 49.0 |
| Maldives | 31.0 |
| Malaysia | 28.0 |
| Philippines | 21.0 |
| | None |
| Australia | 52.0 |
| | None |
| France | 85.0 |
| Italy | 64.0 |
| Spain | 31.0 |
| | None |
| Russian Federation | 20.0 |
| | None |
| Unknown | 240.0 |105 different origins, only countries with more than 20 accessions are represented in the graph
NB see note in text of article on the accessions of « unknown » origin

## Slide 2
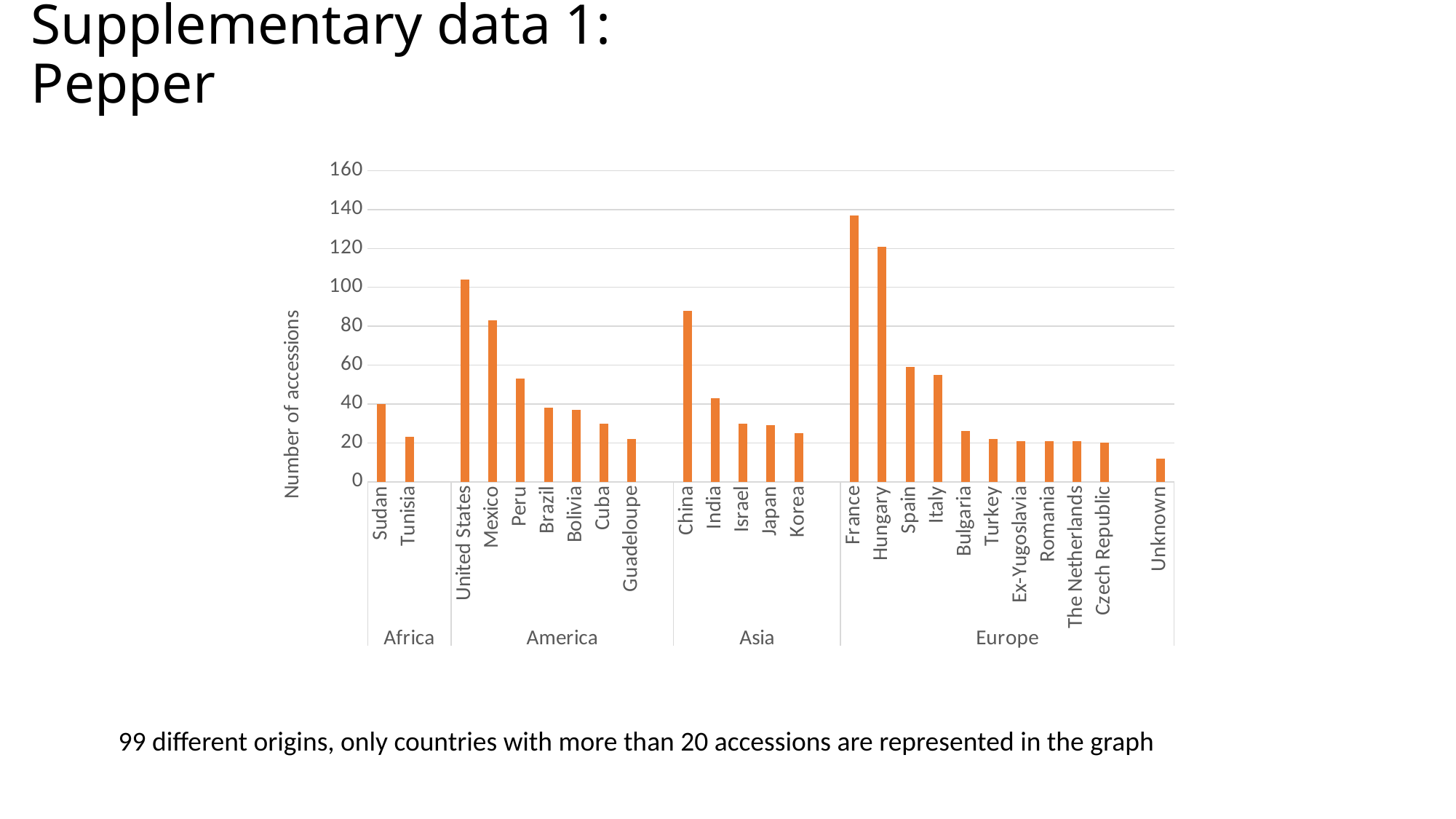

# Supplementary data 1: Pepper
### Chart
| Category | |
|---|---|
| Sudan | 40.0 |
| Tunisia | 23.0 |
| | None |
| United States | 104.0 |
| Mexico | 83.0 |
| Peru | 53.0 |
| Brazil | 38.0 |
| Bolivia | 37.0 |
| Cuba | 30.0 |
| Guadeloupe | 22.0 |
| | None |
| China | 88.0 |
| India | 43.0 |
| Israel | 30.0 |
| Japan | 29.0 |
| Korea | 25.0 |
| | None |
| France | 137.0 |
| Hungary | 121.0 |
| Spain | 59.0 |
| Italy | 55.0 |
| Bulgaria | 26.0 |
| Turkey | 22.0 |
| Ex-Yugoslavia | 21.0 |
| Romania | 21.0 |
| The Netherlands | 21.0 |
| Czech Republic | 20.0 |
| | None |
| Unknown | 12.0 |99 different origins, only countries with more than 20 accessions are represented in the graph

## Slide 3
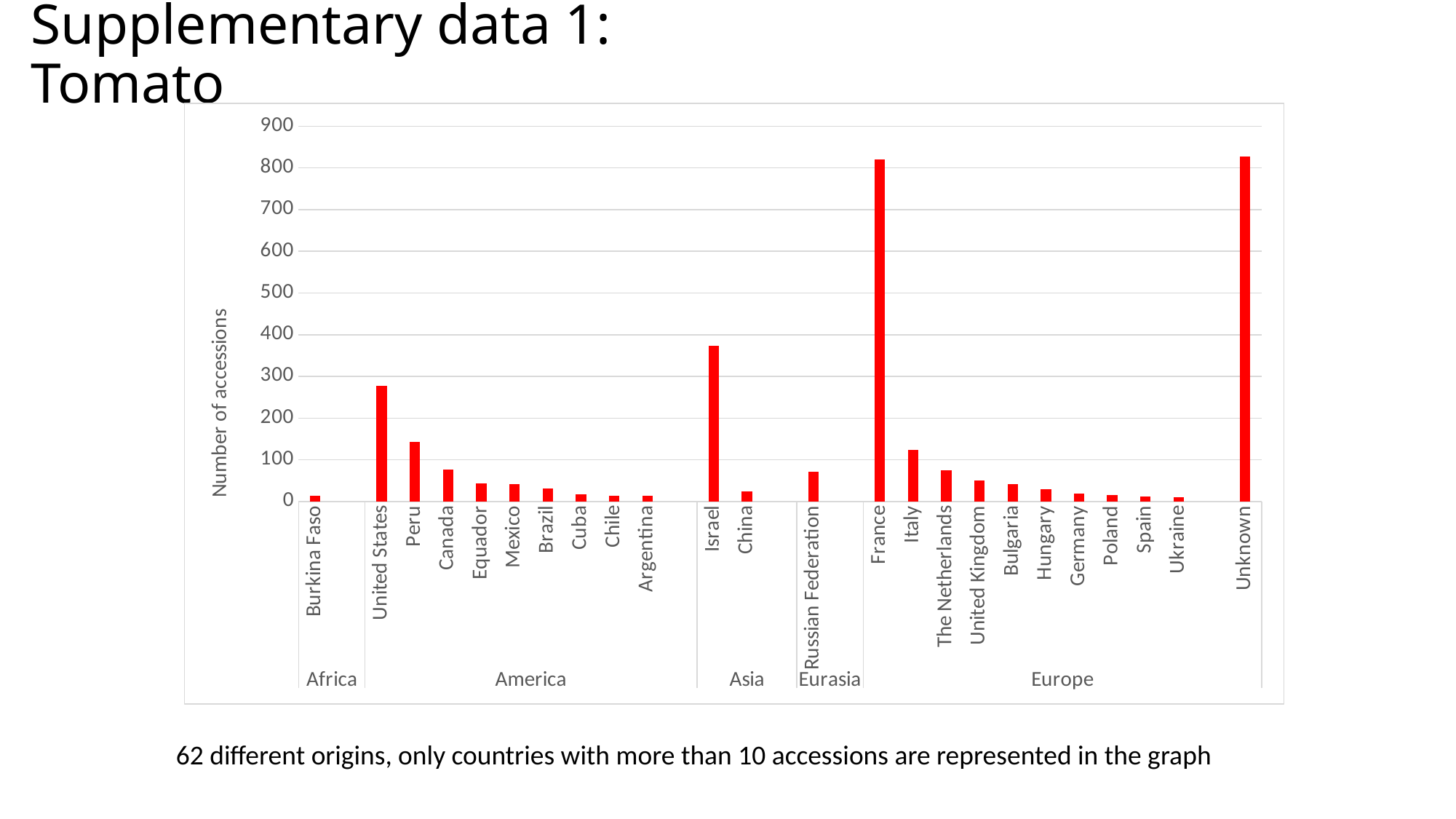

# Supplementary data 1: Tomato
### Chart
| Category | |
|---|---|
| Burkina Faso | 13.0 |
| | None |
| United States | 277.0 |
| Peru | 143.0 |
| Canada | 77.0 |
| Equador | 43.0 |
| Mexico | 41.0 |
| Brazil | 32.0 |
| Cuba | 18.0 |
| Chile | 14.0 |
| Argentina | 13.0 |
| | None |
| Israel | 374.0 |
| China | 25.0 |
| | None |
| Russian Federation | 71.0 |
| | None |
| France | 820.0 |
| Italy | 123.0 |
| The Netherlands | 74.0 |
| United Kingdom | 50.0 |
| Bulgaria | 42.0 |
| Hungary | 30.0 |
| Germany | 19.0 |
| Poland | 15.0 |
| Spain | 12.0 |
| Ukraine | 11.0 |
| | None |
| Unknown | 828.0 |62 different origins, only countries with more than 10 accessions are represented in the graph

## Slide 4
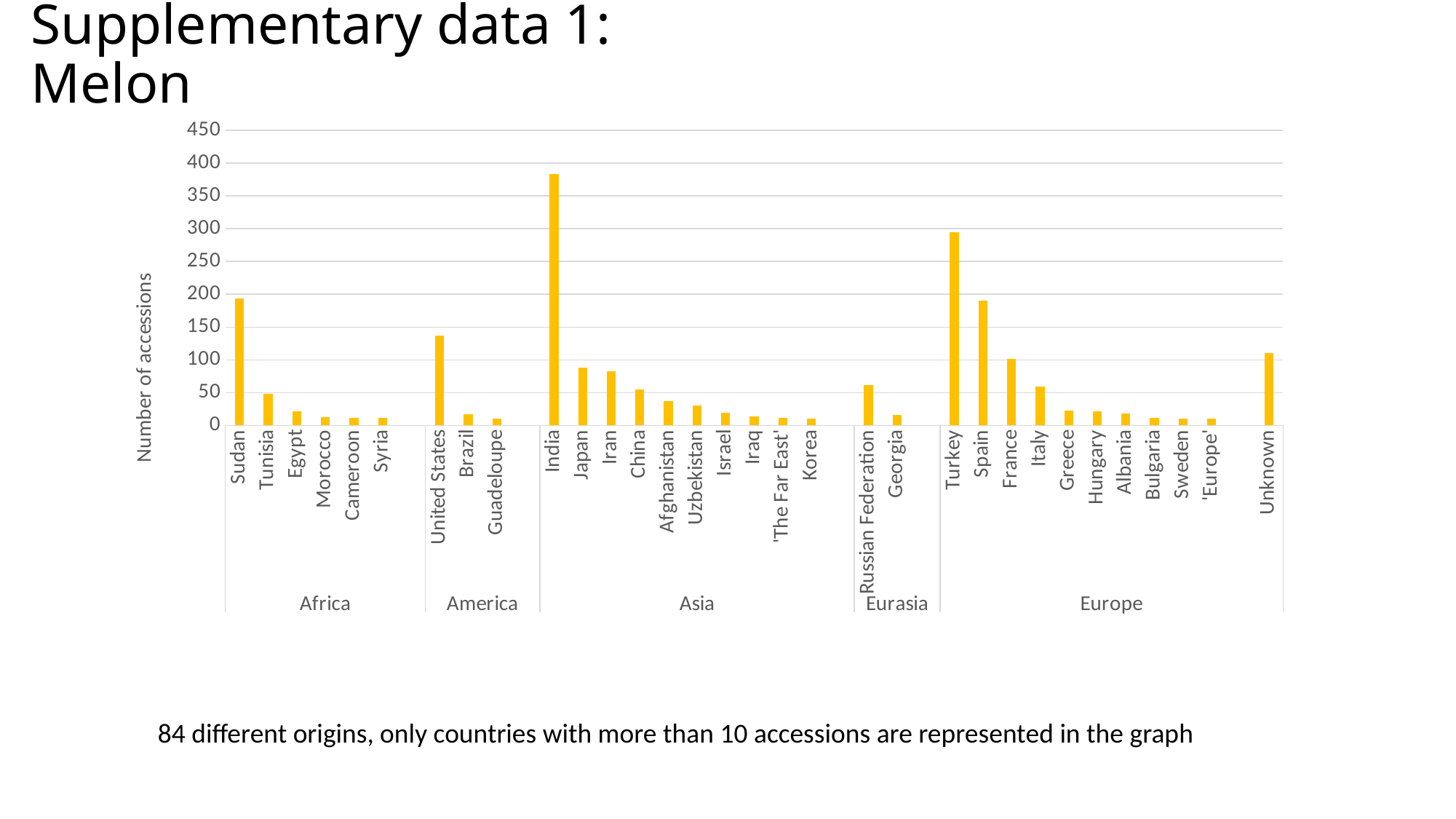

# Supplementary data 1: Melon
### Chart
| Category | |
|---|---|
| Sudan | 194.0 |
| Tunisia | 48.0 |
| Egypt | 22.0 |
| Morocco | 13.0 |
| Cameroon | 12.0 |
| Syria | 11.0 |
| | None |
| United States | 137.0 |
| Brazil | 17.0 |
| Guadeloupe | 10.0 |
| | None |
| India | 383.0 |
| Japan | 88.0 |
| Iran | 82.0 |
| China | 55.0 |
| Afghanistan | 37.0 |
| Uzbekistan | 30.0 |
| Israel | 19.0 |
| Iraq | 14.0 |
| 'The Far East' | 12.0 |
| Korea | 10.0 |
| | None |
| Russian Federation | 61.0 |
| Georgia | 16.0 |
| | None |
| Turkey | 295.0 |
| Spain | 190.0 |
| France | 101.0 |
| Italy | 59.0 |
| Greece | 23.0 |
| Hungary | 22.0 |
| Albania | 18.0 |
| Bulgaria | 11.0 |
| Sweden | 10.0 |
| 'Europe' | 10.0 |
| | None |
| Unknown | 110.0 |84 different origins, only countries with more than 10 accessions are represented in the graph

## Slide 5
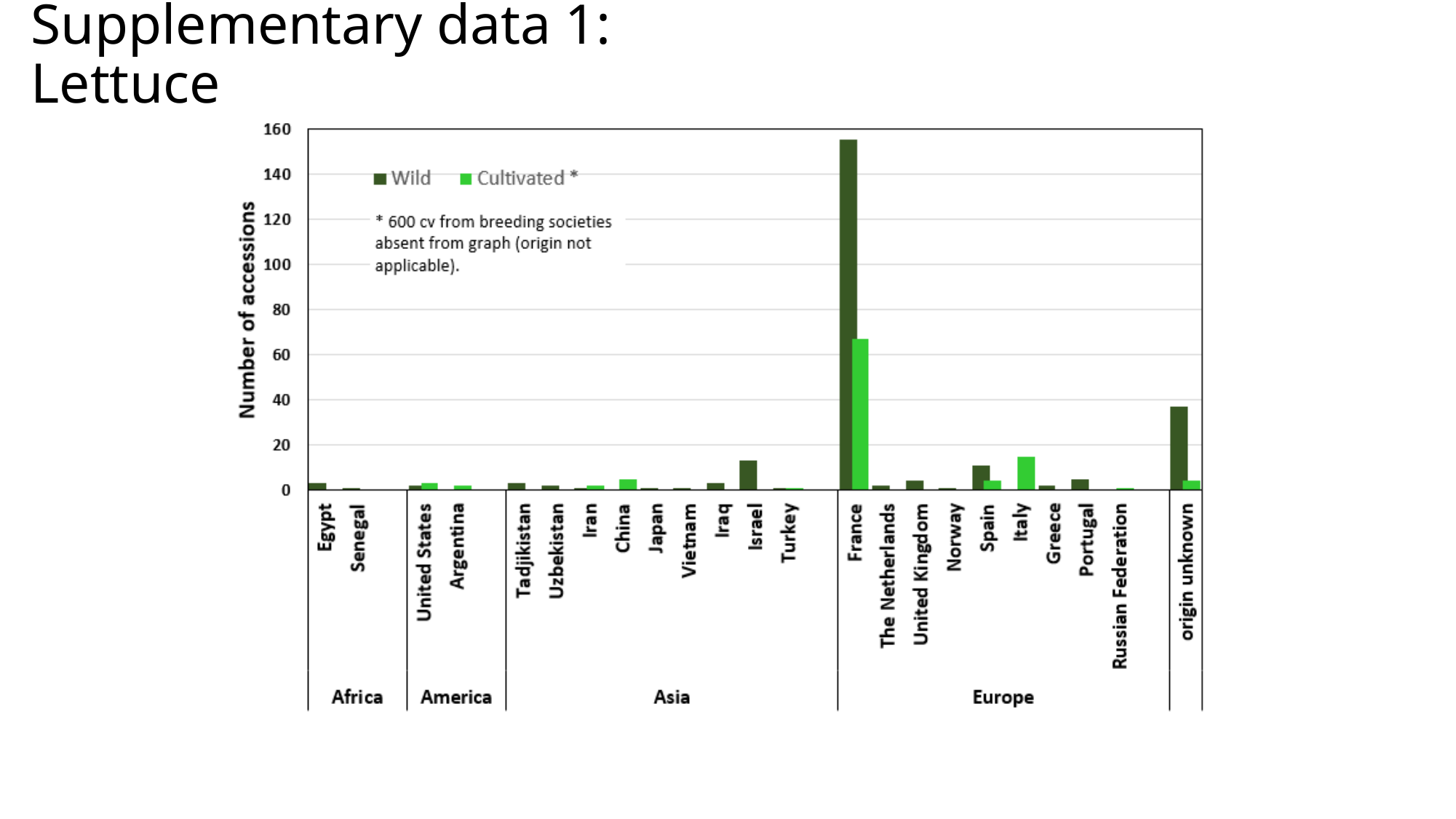

# Supplementary data 1: Lettuce

## Slide 6
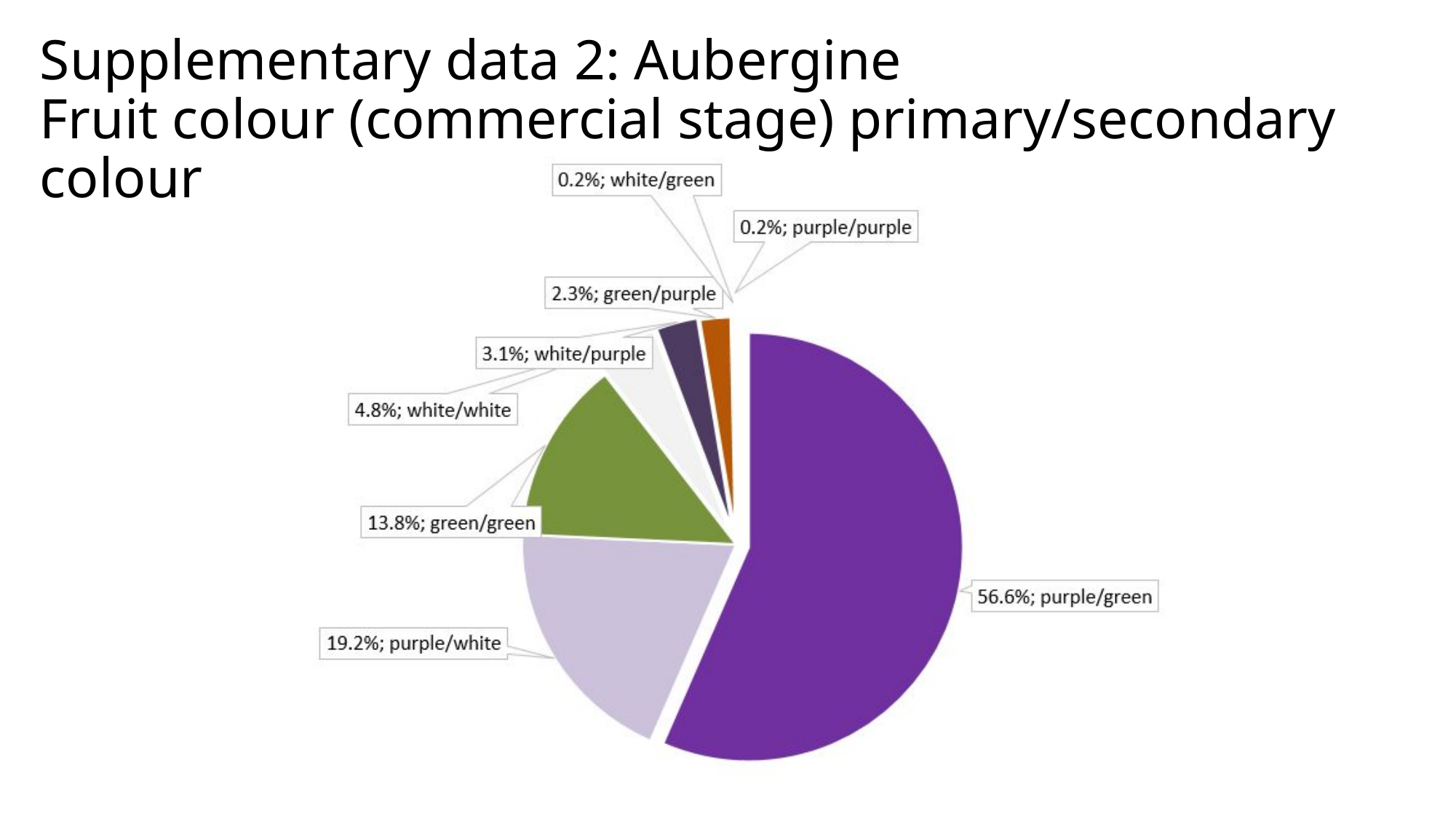

Supplementary data 2: Aubergine
Fruit colour (commercial stage) primary/secondary colour

## Slide 7
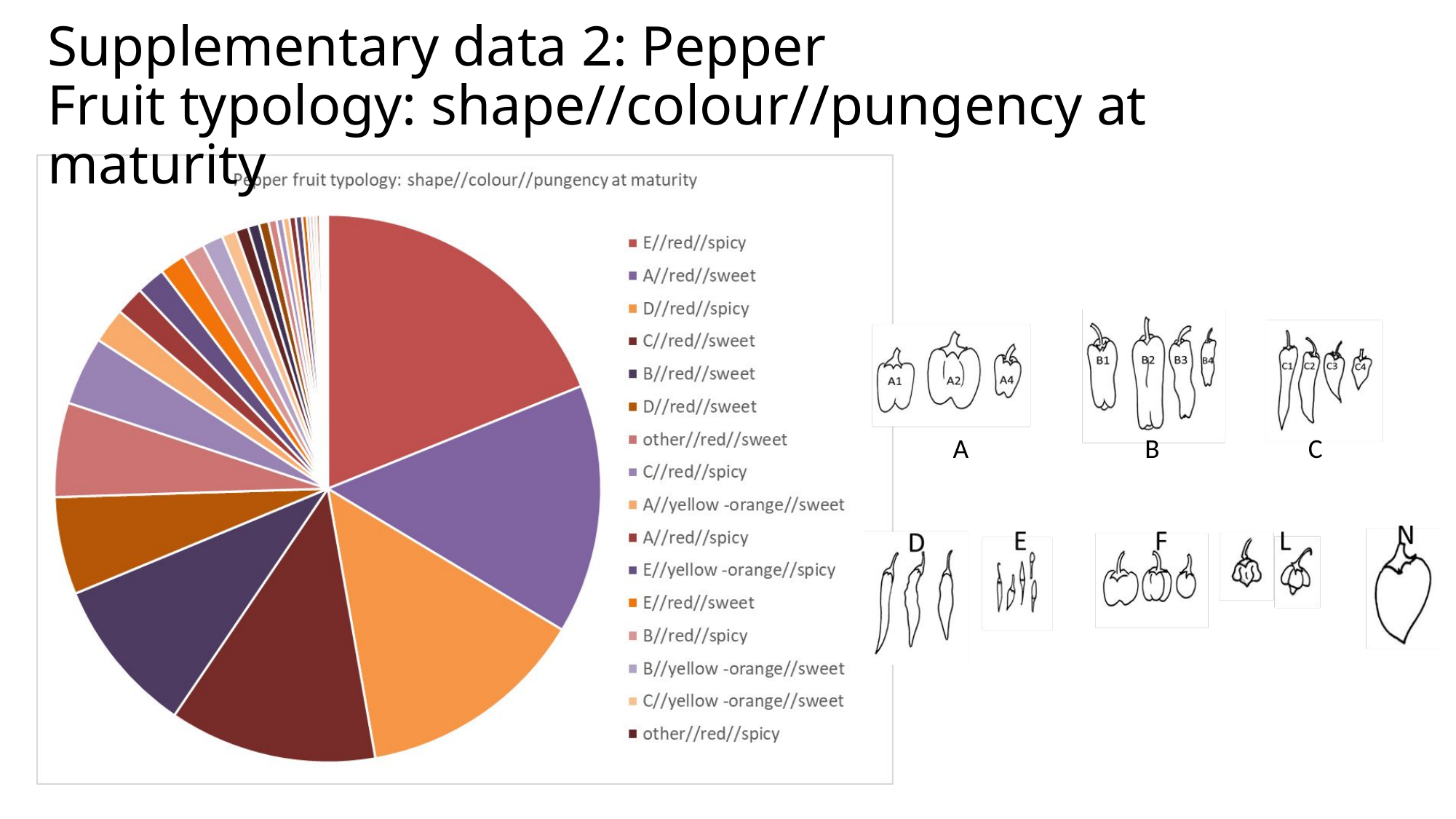

Supplementary data 2: Pepper
Fruit typology: shape//colour//pungency at maturity
A
B
C

## Slide 8
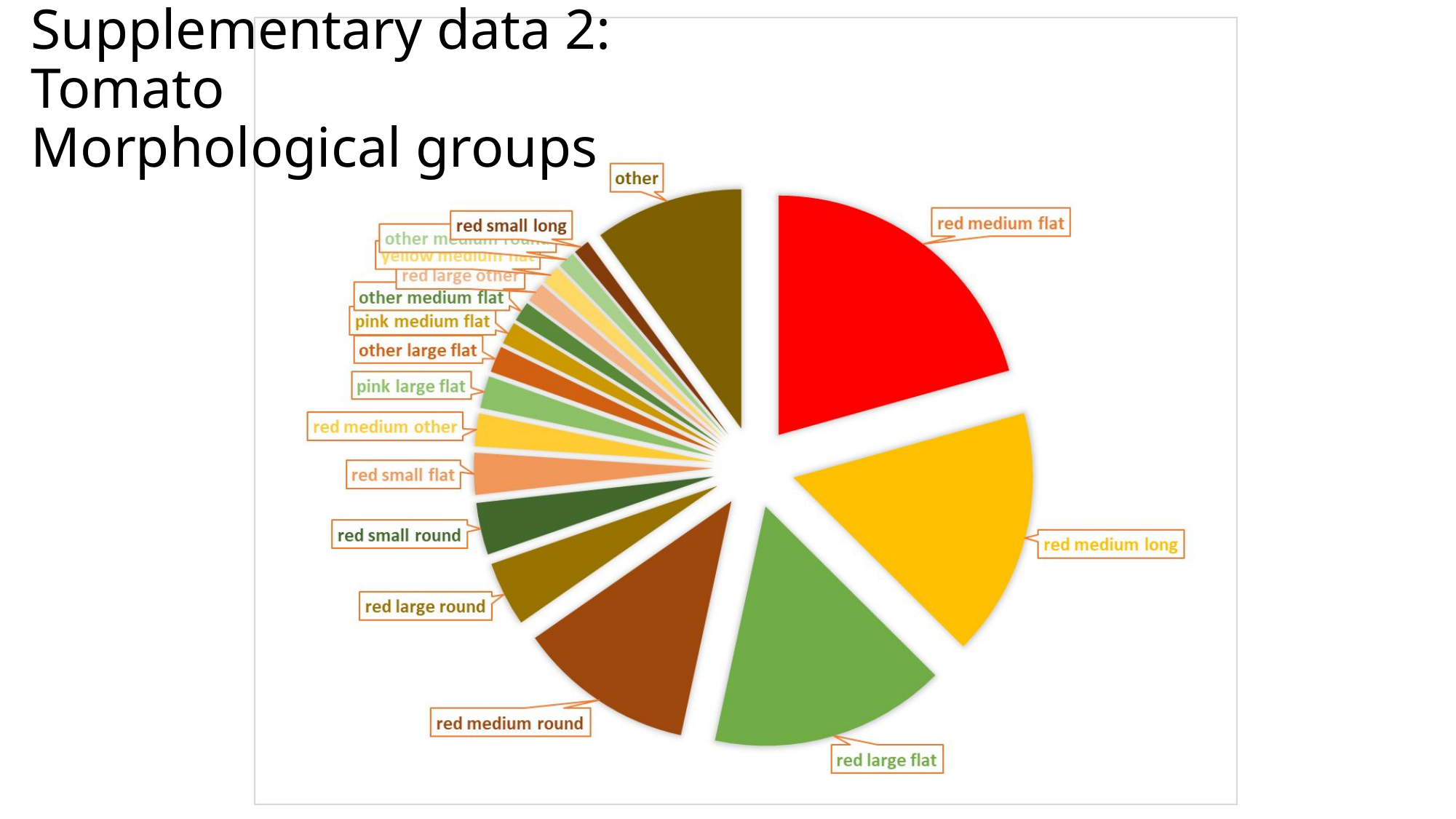

# Supplementary data 2: Tomato Morphological groups

## Slide 9
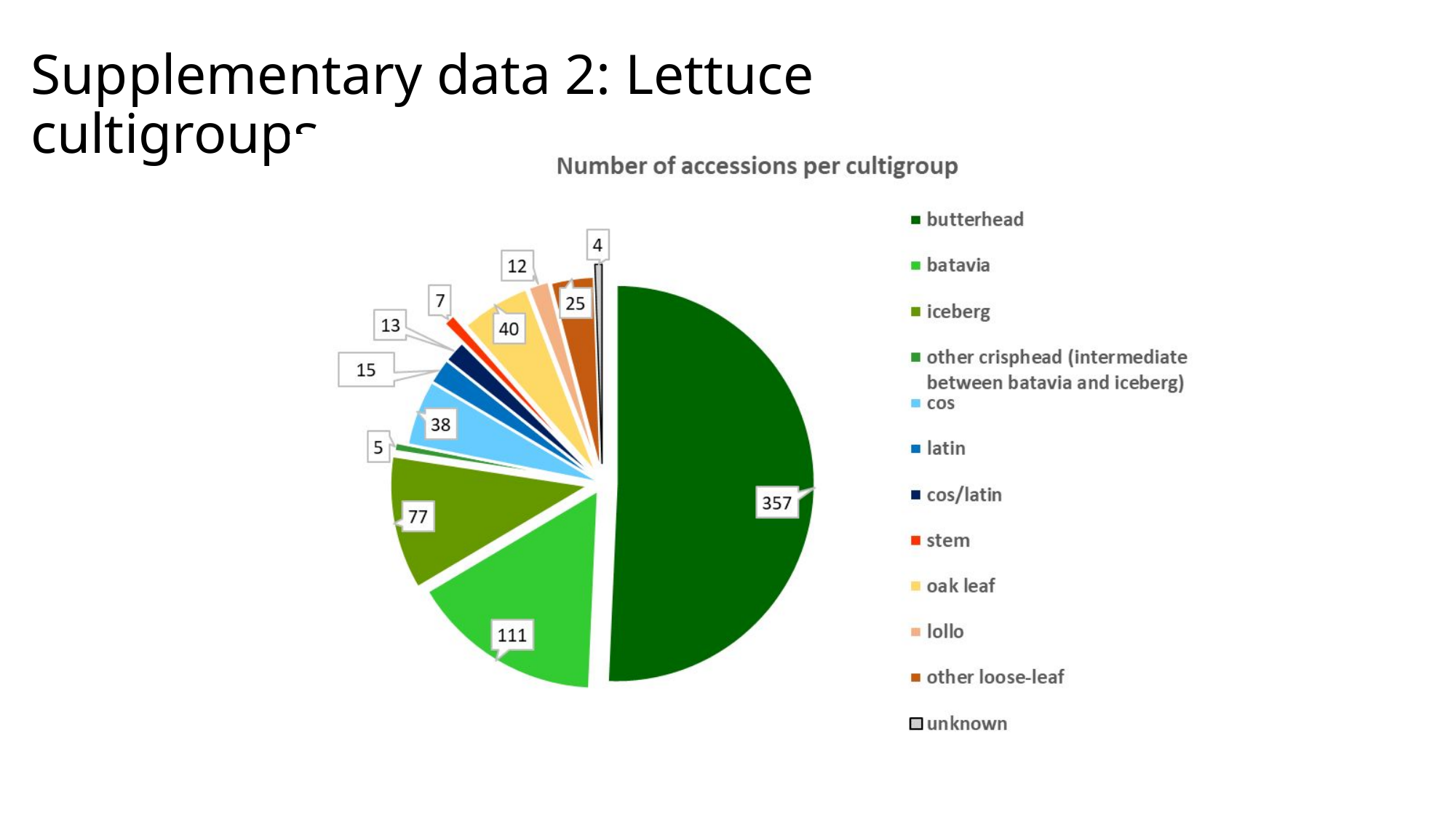

Supplementary data 2: Lettuce cultigroups

## Slide 10
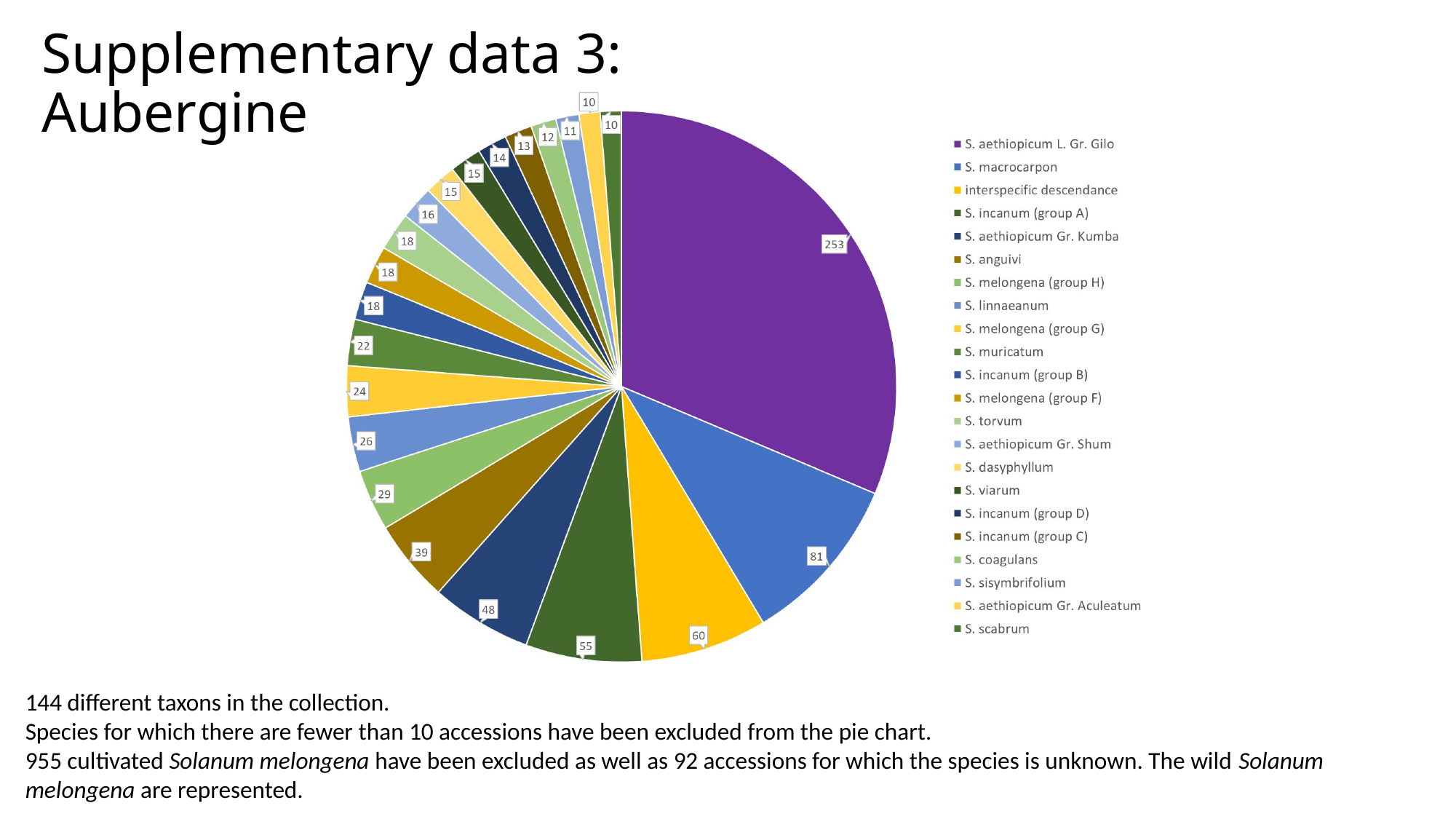

Supplementary data 3:
Aubergine
144 different taxons in the collection.
Species for which there are fewer than 10 accessions have been excluded from the pie chart.
955 cultivated Solanum melongena have been excluded as well as 92 accessions for which the species is unknown. The wild Solanum melongena are represented.

## Slide 11
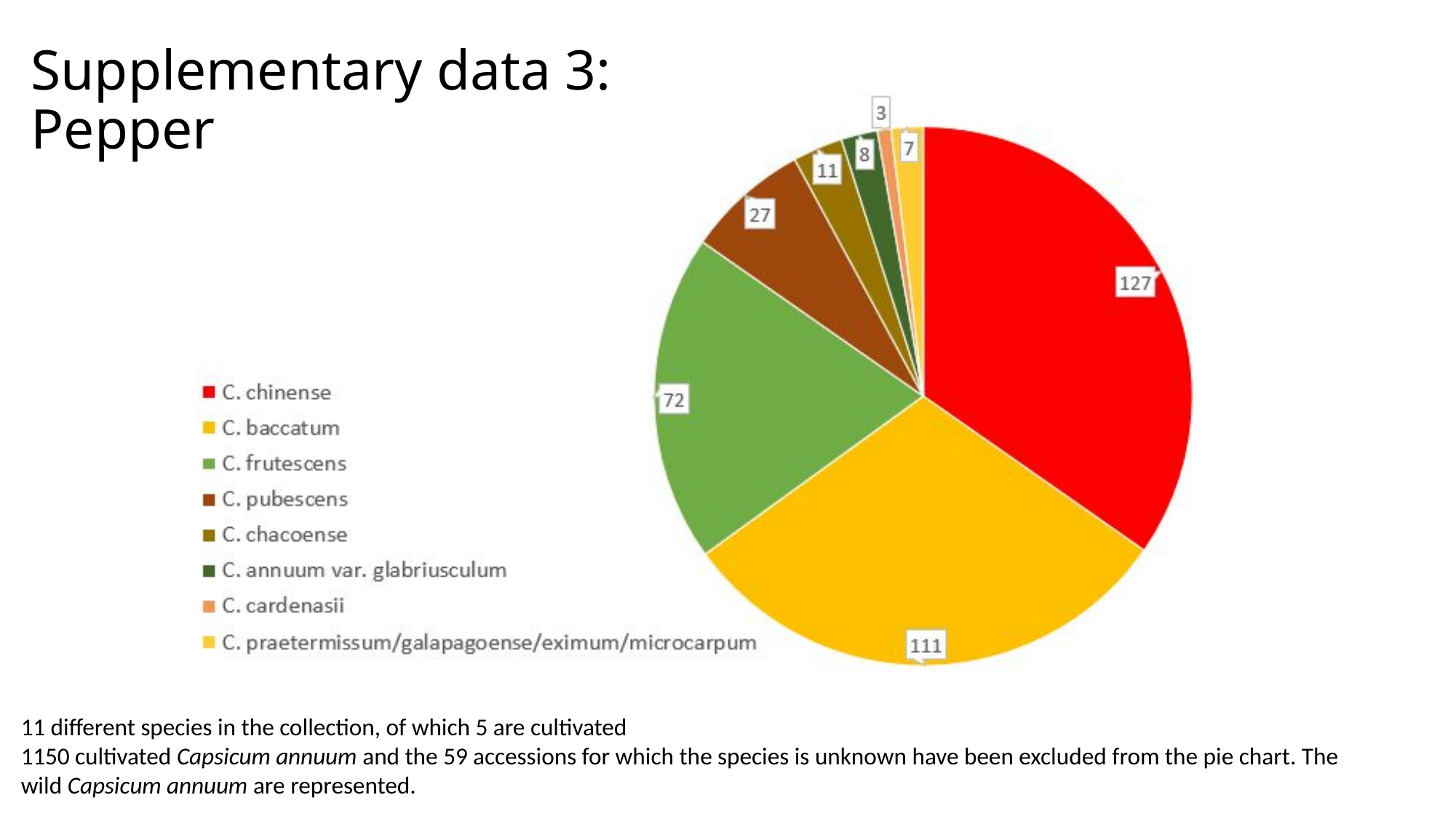

Supplementary data 3: Pepper
11 different species in the collection, of which 5 are cultivated
1150 cultivated Capsicum annuum and the 59 accessions for which the species is unknown have been excluded from the pie chart. The wild Capsicum annuum are represented.

## Slide 12
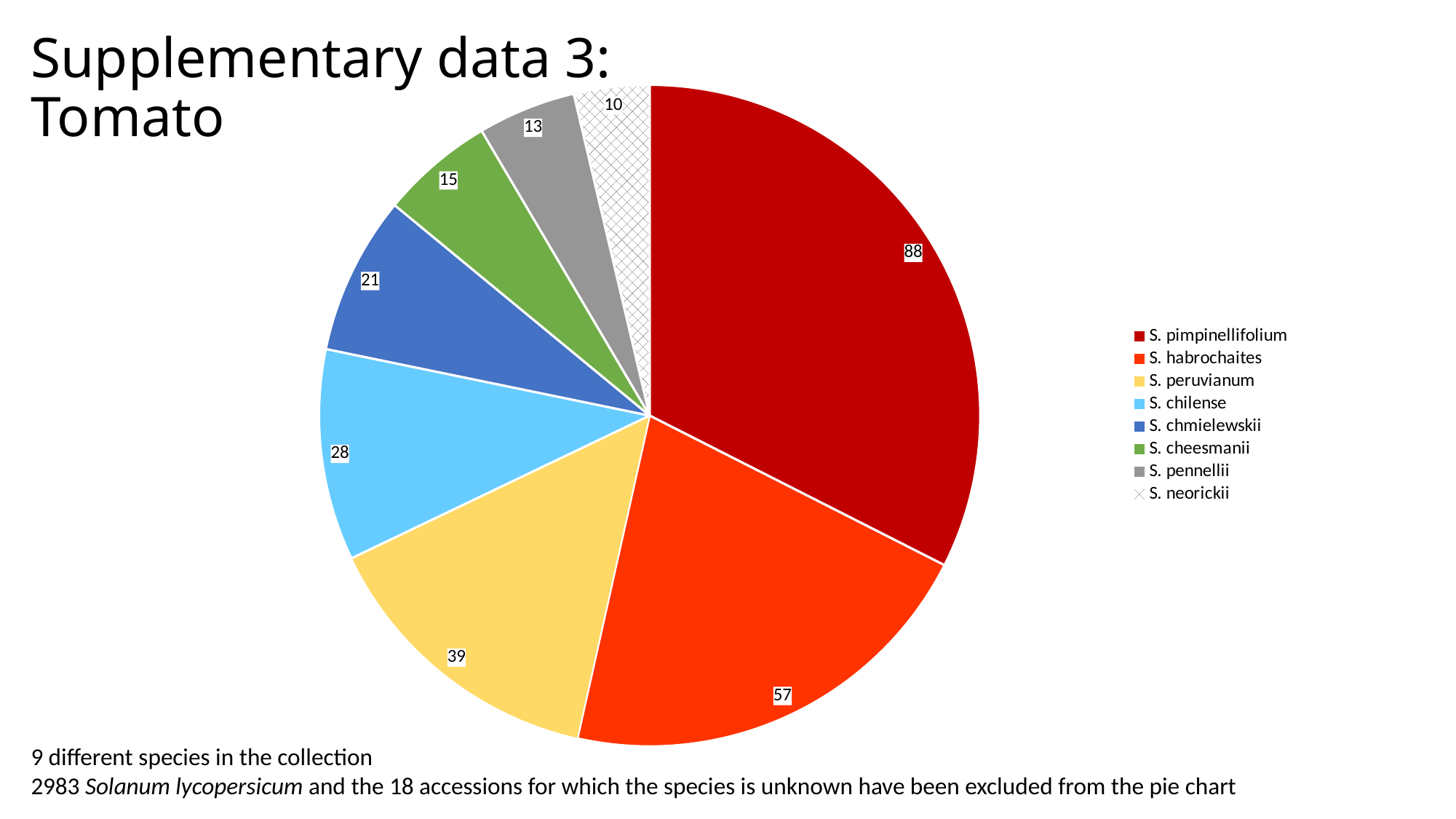

Supplementary data 3: Tomato
### Chart
| Category | |
|---|---|
| S. pimpinellifolium | 88.0 |
| S. habrochaites | 57.0 |
| S. peruvianum | 39.0 |
| S. chilense | 28.0 |
| S. chmielewskii | 21.0 |
| S. cheesmanii | 15.0 |
| S. pennellii | 13.0 |
| S. neorickii | 10.0 |9 different species in the collection
2983 Solanum lycopersicum and the 18 accessions for which the species is unknown have been excluded from the pie chart

## Slide 13
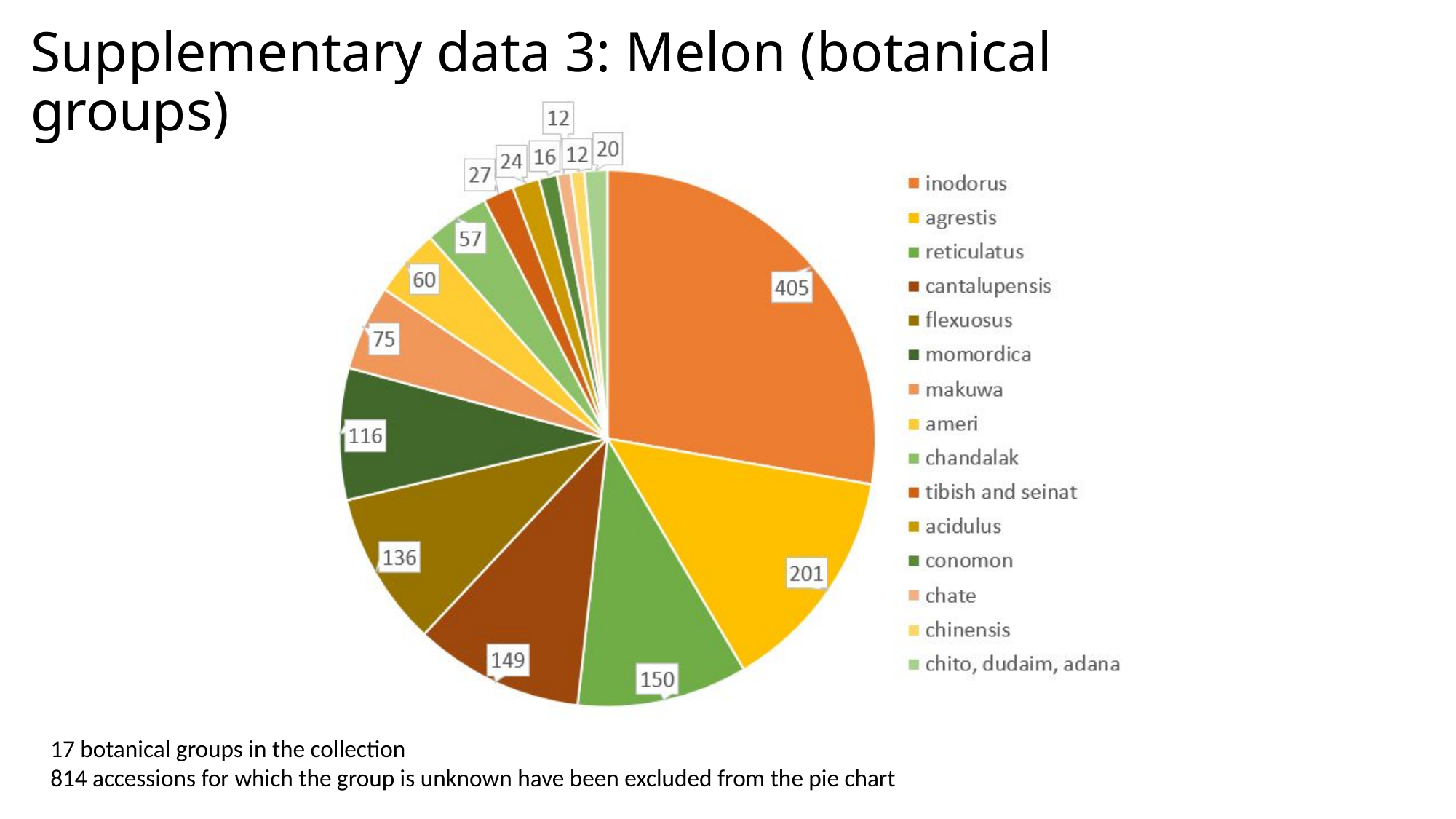

Supplementary data 3: Melon (botanical groups)
17 botanical groups in the collection
814 accessions for which the group is unknown have been excluded from the pie chart

## Slide 14
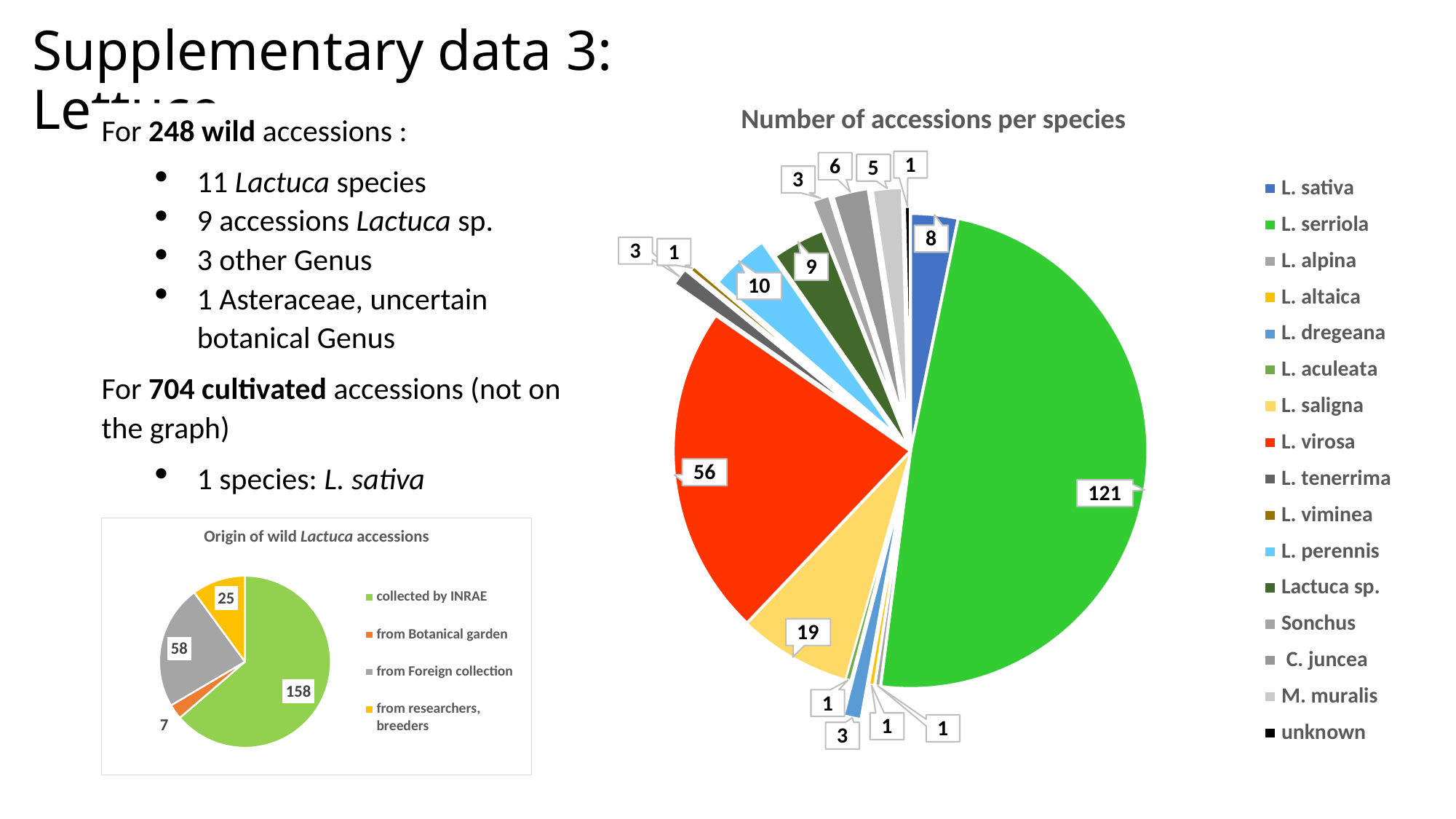

Supplementary data 3: Lettuce
For 248 wild accessions :
11 Lactuca species
9 accessions Lactuca sp.
3 other Genus
1 Asteraceae, uncertain botanical Genus
For 704 cultivated accessions (not on the graph)
1 species: L. sativa

## Slide 15
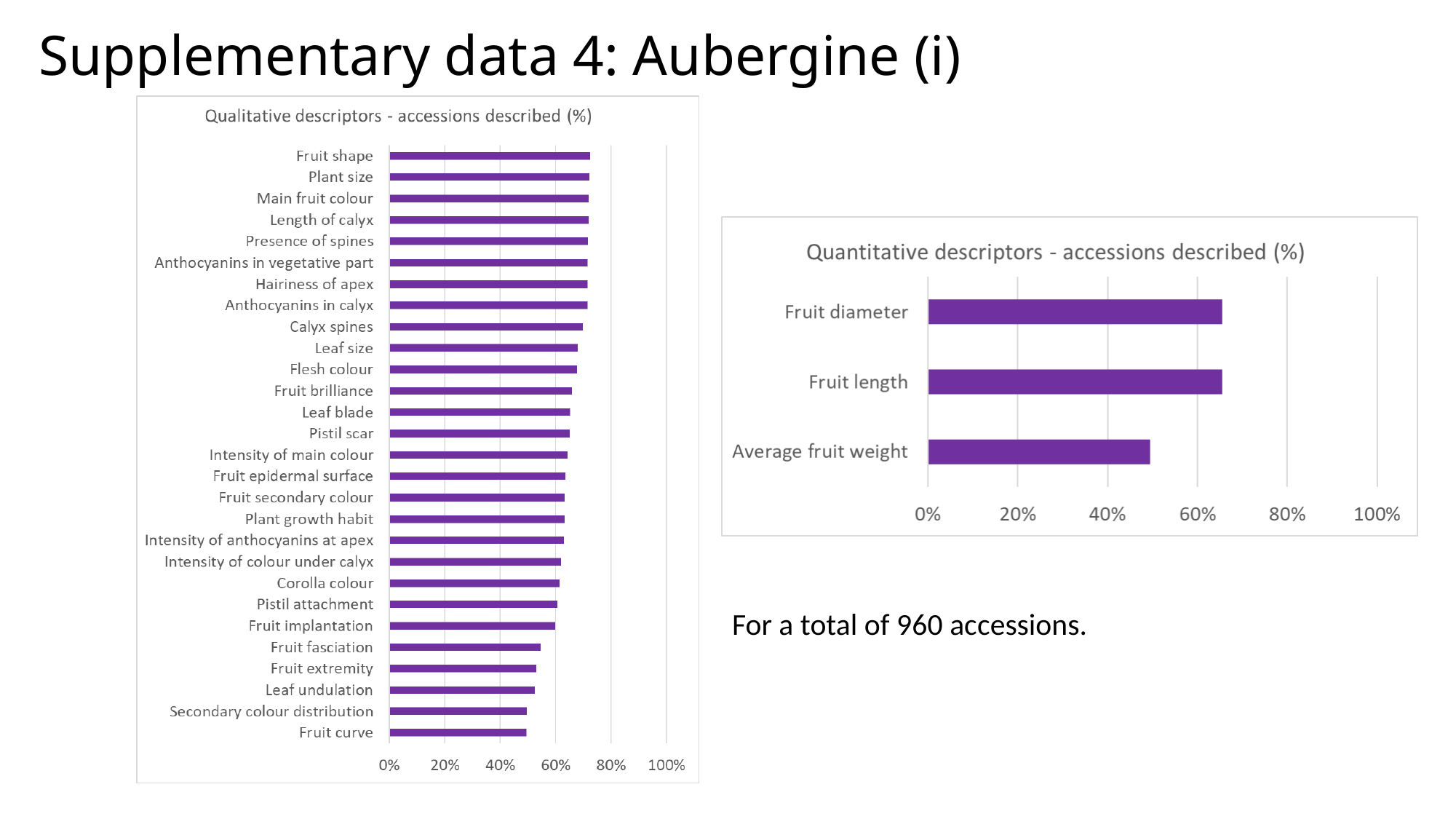

Supplementary data 4: Aubergine (i)
For a total of 960 accessions.

## Slide 16
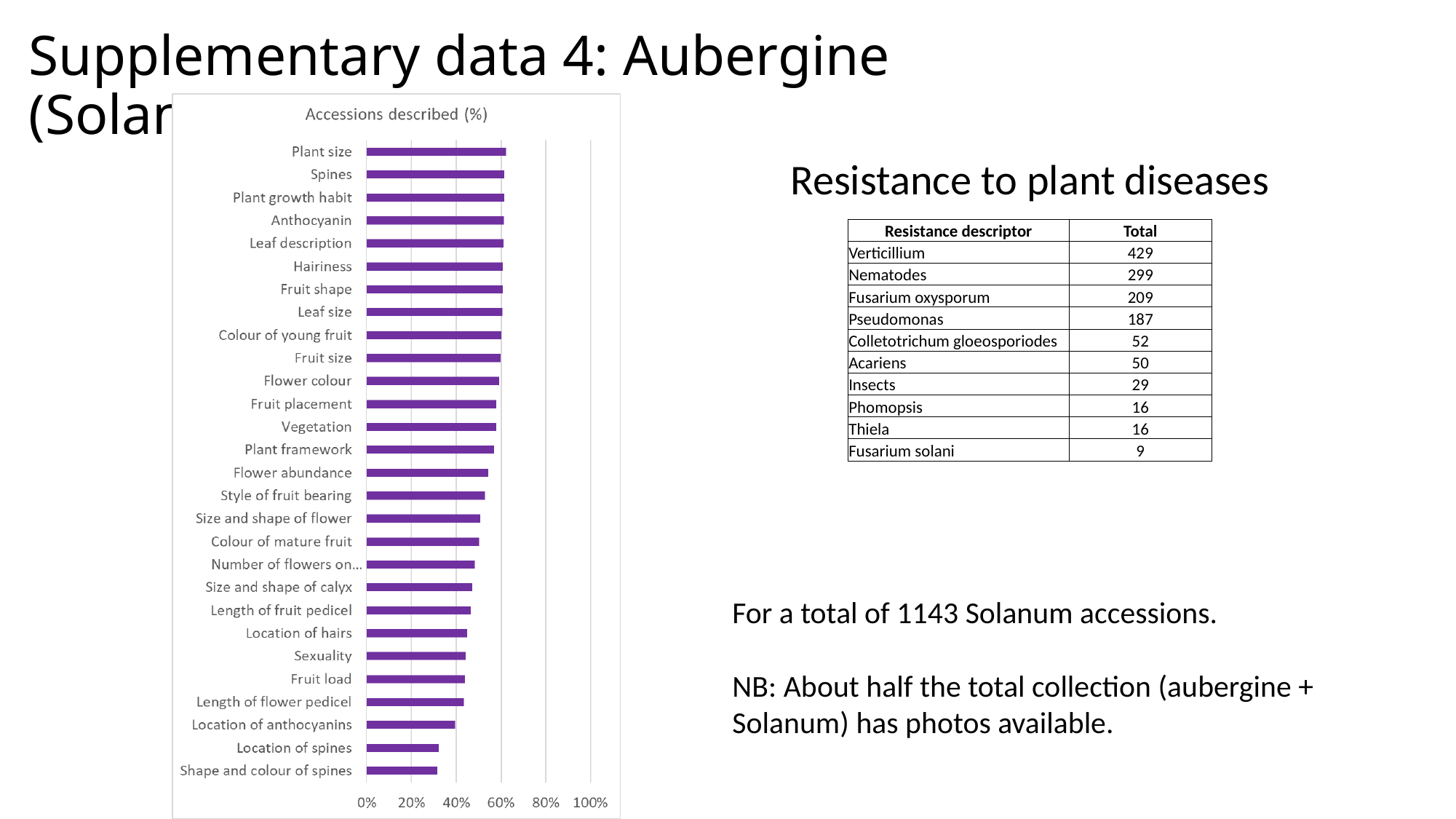

Supplementary data 4: Aubergine (Solanum)
Resistance to plant diseases
| Resistance descriptor | Total |
| --- | --- |
| Verticillium | 429 |
| Nematodes | 299 |
| Fusarium oxysporum | 209 |
| Pseudomonas | 187 |
| Colletotrichum gloeosporiodes | 52 |
| Acariens | 50 |
| Insects | 29 |
| Phomopsis | 16 |
| Thiela | 16 |
| Fusarium solani | 9 |
For a total of 1143 Solanum accessions.
NB: About half the total collection (aubergine + Solanum) has photos available.

## Slide 17
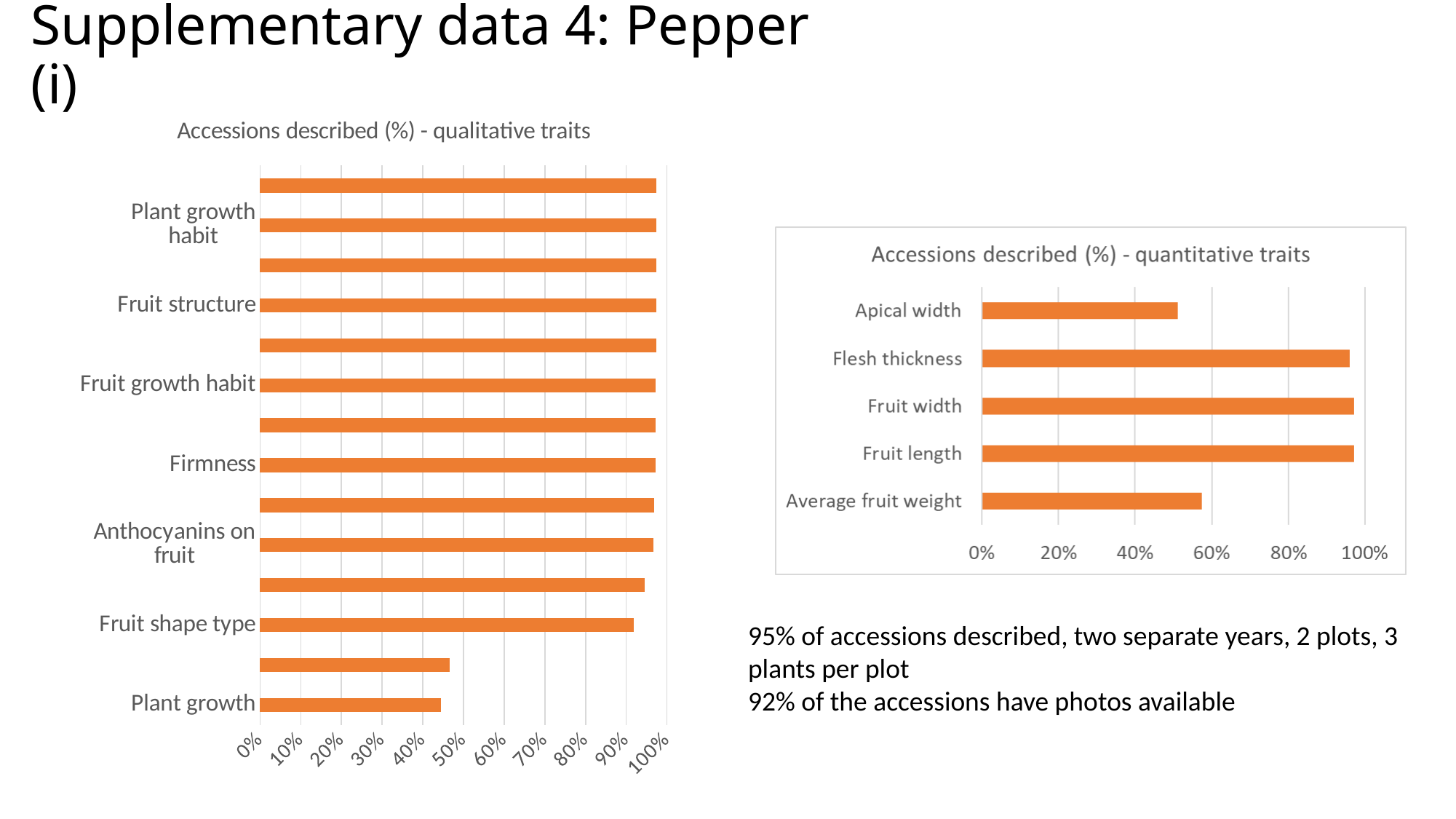

Supplementary data 4: Pepper (i)
### Chart: Accessions described (%) - qualitative traits
| Category | % renseigné |
|---|---|
| Plant growth | 0.4447284345047923 |
| Roots | 0.46645367412140576 |
| Fruit shape type | 0.9175718849840255 |
| Fruit aspect | 0.9444089456869009 |
| Anthocyanins on fruit | 0.9667731629392972 |
| Hairiness | 0.9693290734824281 |
| Firmness | 0.9718849840255591 |
| Capsaicin presence | 0.9718849840255591 |
| Fruit growth habit | 0.9725239616613418 |
| Fruit colour at maturity | 0.9731629392971246 |
| Fruit structure | 0.9731629392971246 |
| Leaf size | 0.9738019169329073 |
| Plant growth habit | 0.9744408945686901 |
| Fruit colour before maturity | 0.9744408945686901 |
95% of accessions described, two separate years, 2 plots, 3 plants per plot
92% of the accessions have photos available

## Slide 18
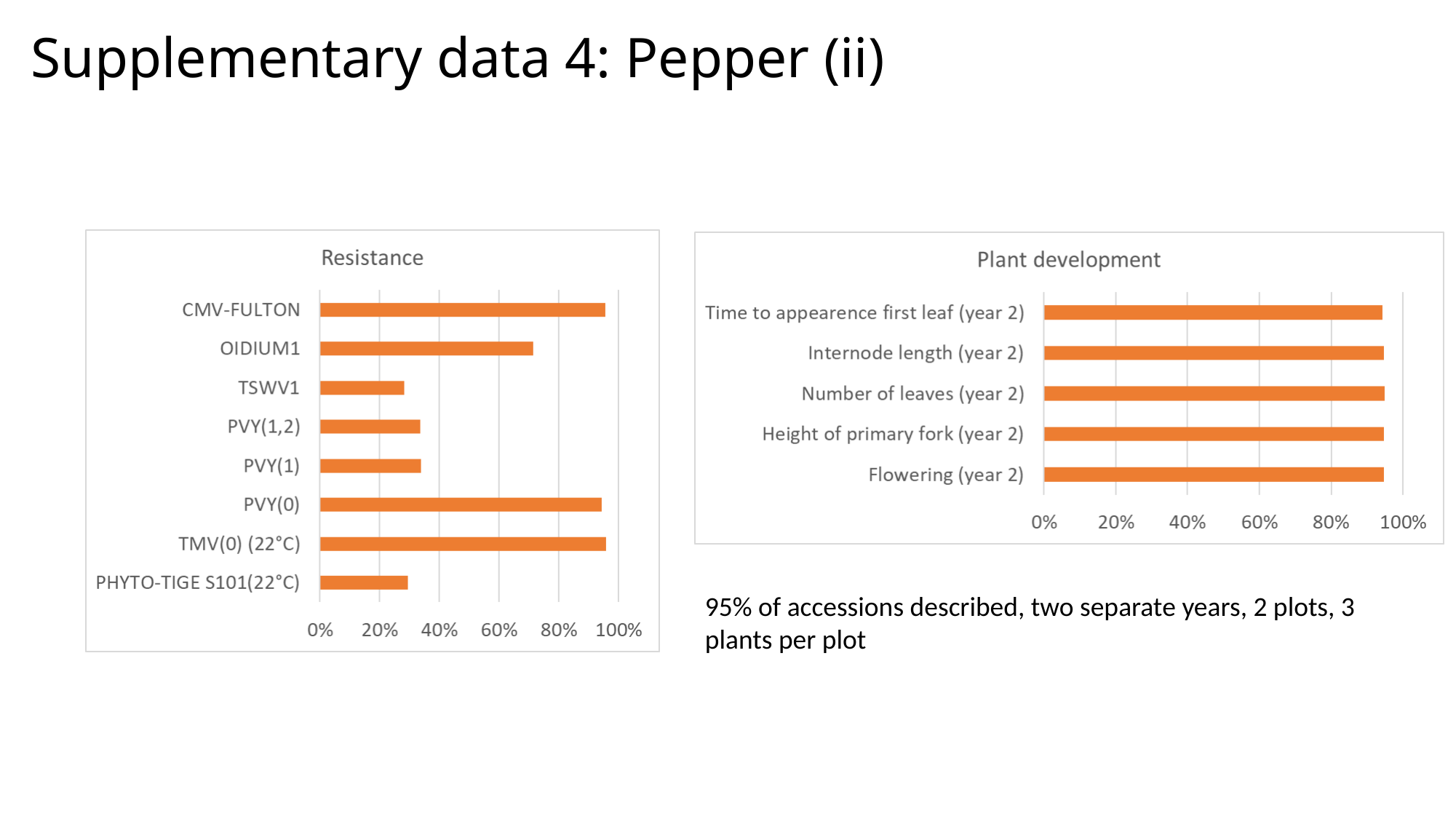

Supplementary data 4: Pepper (ii)
95% of accessions described, two separate years, 2 plots, 3 plants per plot

## Slide 19
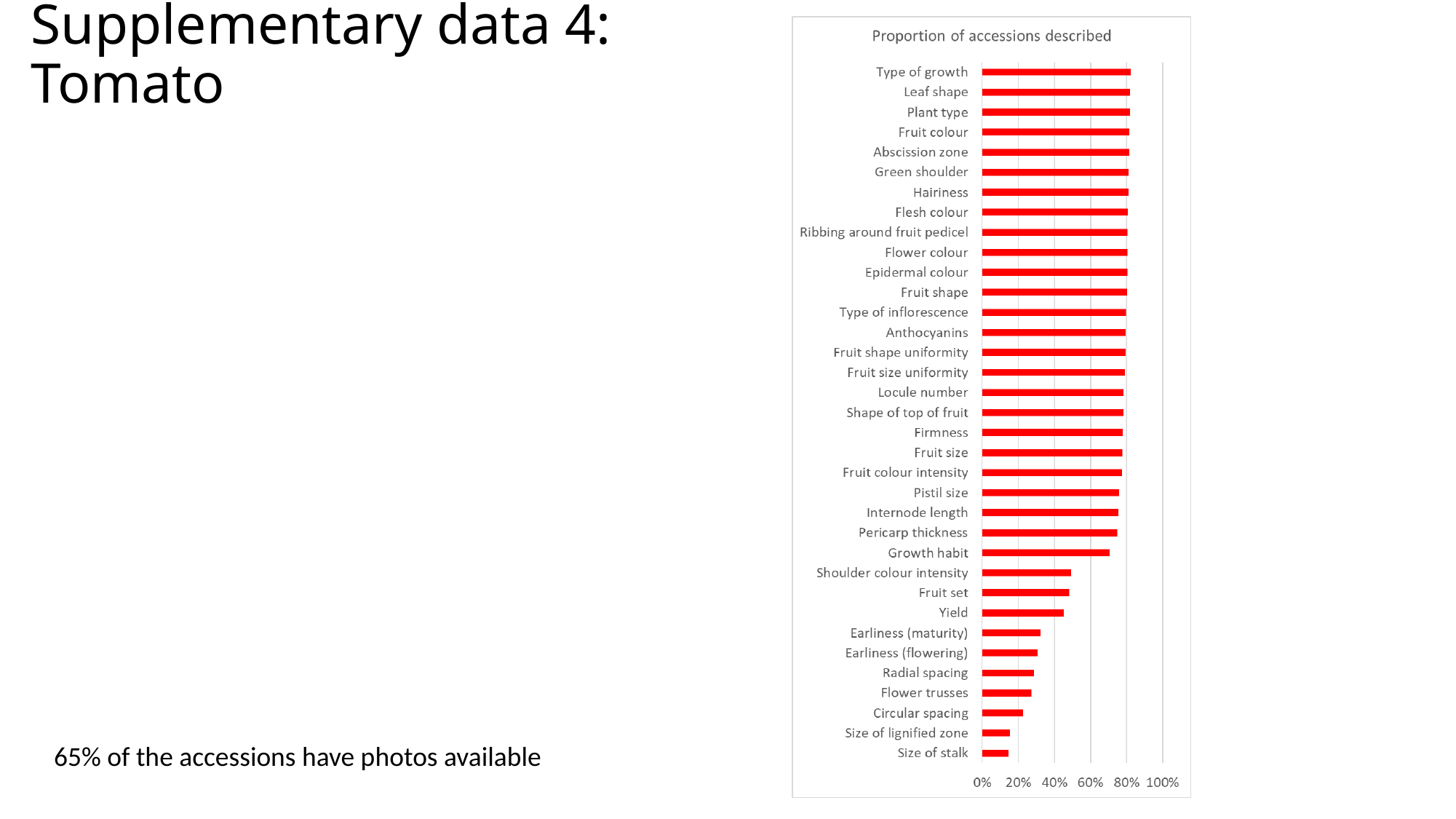

Supplementary data 4: Tomato
65% of the accessions have photos available

## Slide 20
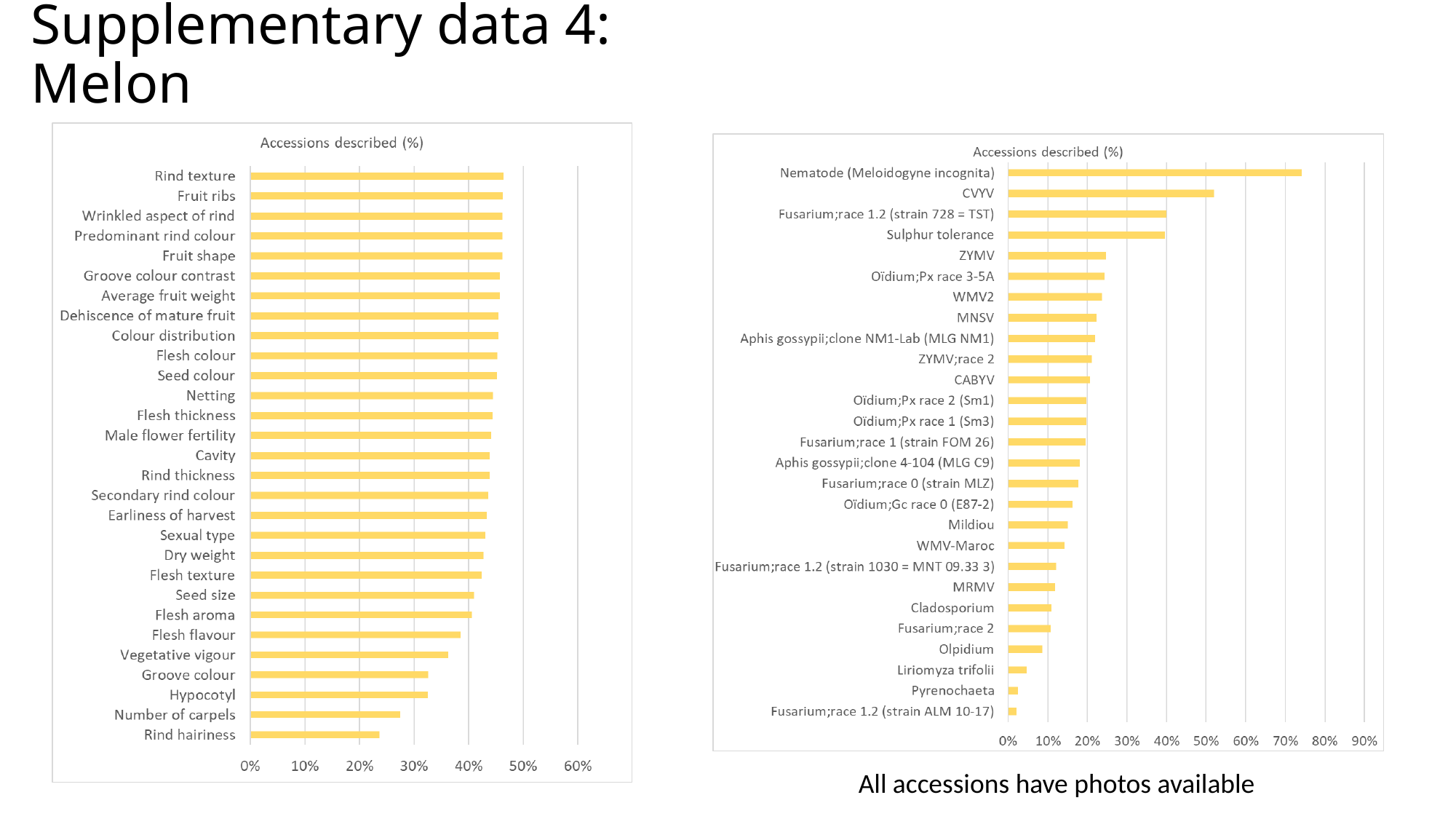

Supplementary data 4: Melon
All accessions have photos available

## Slide 21
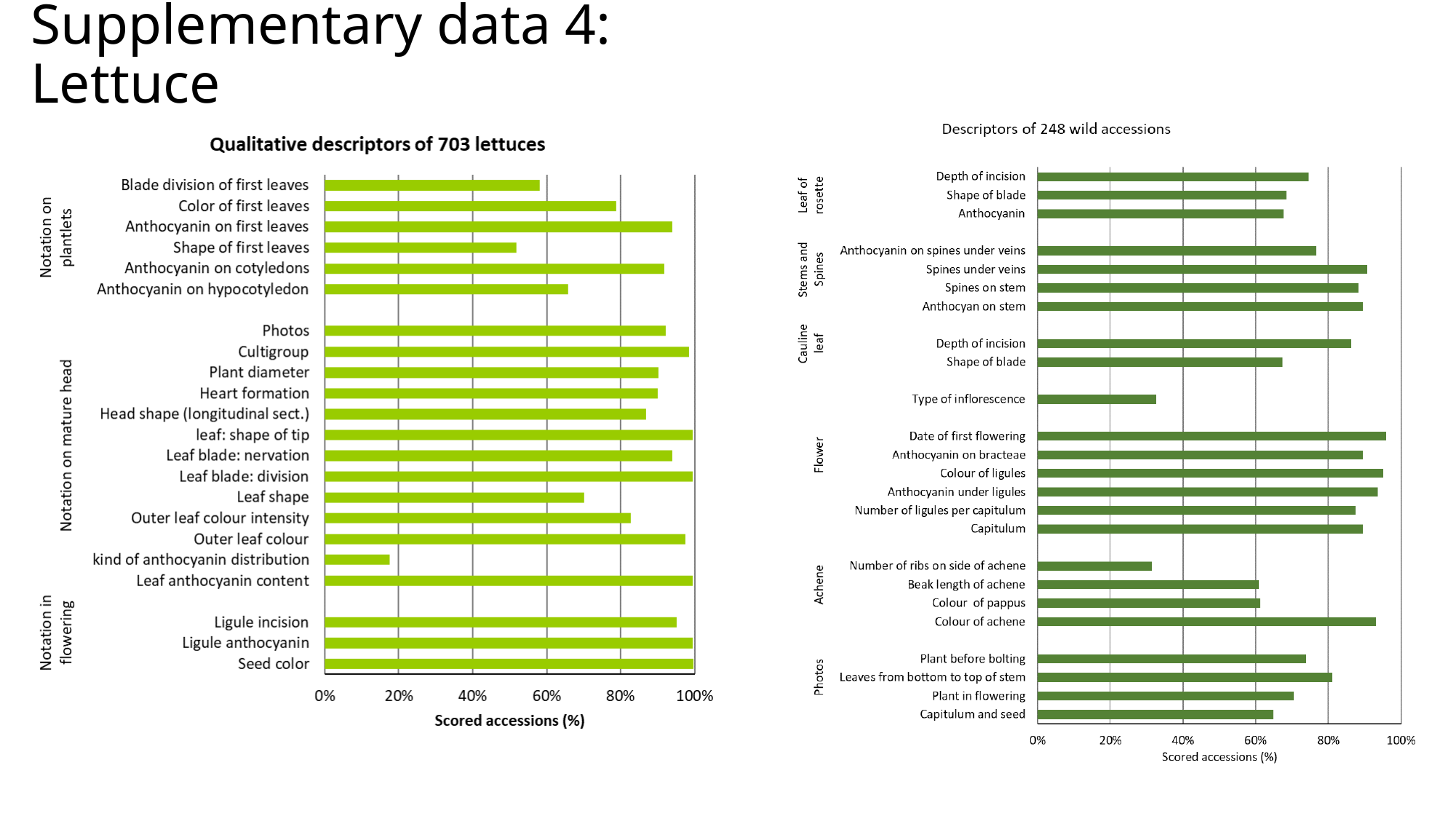

Supplementary data 4: Lettuce
