# Supplementary material for: The INRAE Centre for Vegetable Germplasm: Geographically and Phenotypically Diverse Collections and Their Use in Genetics and Plant Breeding
Source: Plants (Basel). 2022 Jan 27;11(3):347. doi: 10.3390/plants11030347 (PMC8838894; doi:10.3390/plants11030347)
Supplement: Supplementary file 1 [file plants-11-00347-s001.zip › suppTableI_SalinierEtAl.pdf]

Supplementary Table SI: Non-exhaustive list of Vegetable Genebanks worldwide.

Genebank	Location	Website	Accessions and species
Scientific Center of Vegetable & Industrial Crops	Darakert, Armenia		Includes melon
AGES, Pflanzengenetische Ressourcen	Linz, Austria	https://www.genbank.at/en.html	
Azerbaijan National Academy of Science, Institute of Genetic Resources	Baku, Azerbaijan	https://science.gov.az/en/institutes/338	7355 samples of seeds; 871 species.
BGH, Vegetable Germplasm Bank	Universidade Federal de Viçosa, Viçosa, Brazil		8,000 vegetables accessions. Priorities: ancient varieties, selections made by farmers, variant forms of cultivated species and wild species of the interest genera to the national horticulture. Solanaceae (44%), Leguminosae (17%) and Curcubitaceae (16%).
Brazilian Agricultural Research Corporation (Embrapa)	Brasilia, Brazil		[1]
Institute for Plant Genetic Resources	Sadovo, Bulgaria		60,000 seeds of 2,670 different plant species.
Plant Gene Resources of Canada	Saskatoon, Canada		Includes cereals, pulses, oilseeds, tomato, forages and covers many species.
Vegetable Genetic Resources in China	Bejing, China		[2]
CRI, Department of Vegetables and Special Crops	Olomouc, Czech Republic		Vegetables, spicy, aromatic and medicinal plants
EBI, Ethiopian Biodiversity Institute	Addis Ababa, Ethiopia		87,000 accessions of crop and forest germplasm. There are about 78 crop species in the genebank.

INRAE Centre for Vegetable Germplasm (CRB-Légumes)	Avignon, France		10,000 accessions of Solanaceae, melon and lettuce
Carrot and other vegetable Apiaceae	Angers, France		Carrot and other vegetable Apiaceae
BrACySol	Ploudaniel, France		Brassicaceae, Alliaceae, Asteraceae, Solanaceae
IPK, Leibniz Institut für Pflanzengenetik und kulturpflanzenforschung	Leibniz, Germany		Totaling 151,002 accessions from 2933 species and 776 genera, the IPK Genebank holds one of the largest collections of crop plants and their wild relatives in the world.
Plant Genetic Resources Research Institute	Bunso, Ghana	http://pgrri.csir.org.gh/	
National Agricultural Research and Innovation Centre, Vegetable Crop Research Department	Gödöllő, Hungary		Breeding of resistant, spicy pepper, tomato, onion and garlic varieties.
National Bureau of Plant Genetic Resources, Indian Council of Agricultural Research (ICAR)	New Delhi, India		Solanaceae: aubergine, tomato, pepper; Cucurbitaceae: pumpkin, melons, gourds and cucumber; Legumes: cowpea, pea, lablab bean, winged bean, faba bean, French bean; Bulb crops: garlic, onion; Root vegetables: radish, carrot, turnip; Also Okra, cole crops, Chinese cabbage, spinach beet, spinach.
IBBR, Institute of Biosciences and BioResources, National Research Council of Italy	Bari, Italy	https://ibbr.cnr.it/ibbr/	At least 3,844 accessions of vegetables (CNR Institutes) [3].
National Agricultural Research Centre	Baq'a, Jordan	http://www.ncare.gov.jo/	
GeRRI, National Genebank of Kenya	Kikuyu, Kenya		50,000 accessions representing over 2,000 plant species.
HHRC, Haddokkeo Horticulture Research Center	Haddokkeo, Laos		Chili pepper, mustard, loofah, aubergine, cucumber, corn, pumpkin, coriander, dill, tomato, lettuce, amaranth.
Department of Agricultural Research	Yezin, Myanmar		

BANGEV, UACH, National Vegetable Germplasm Bank	Chapingo, Mexico		The wild and cultivated species found in Mexico for nutritional, medicinal and comestible use are represented.
CGN, Centre for Genetic Resources	Wageningen University and Research, The Netherlands		13,000 accessions of vegetable crops including lettuce, cabbage and fruit vegetables such as pepper, tomato, cucumber and aubergine.
Faculty of Agriculture, Sciences and Food	Skopje, North Macedonia	http://www.fznh.ukim.edu.mk/en/	
National Institute of Horticultural Research	Skierniewic, Poland	http://www.inhort.pl/en	
Vavilov Institute	St. Petersburg, Russian Federation		[4]
RC-Vegetable & Ornamental Plants	Pretoria, South Africa		Sweet potatoes, indigenous/traditional African vegetables, potatoes, commercial vegetables
CRF, National Plant Genetic Resources Centre	Madrid, Spain		75,000 accessions conserved in the Collections Network of the Spanish Program for the Conservation and Sustainable Use of Plant Genetic Resources for Food and Agriculture (NPPGR).
COMAV, Institute for the Preservation and Improvement of Valencian Agro-diversity	Polytechnic University of Valencia, Spain	http://www.comav.upv.es/	
Plant Genetic Resources for Food and Agriculture	Khartoum, Sudan	http://arcsudan.sd/agricultural-plant-genetic-resources-conservation-and-research-center/	Includes okra, sorghum and banana.

Plant Genetic Resources for Food and Agriculture, Agroscope	Switzerland		759 accessions; includes varieties or accessions which originated or were bred in Switzerland, or were of national, regional or local importance and are not (or no longer) protected by intellectual property rights.
World Vegetable genebank (formerly AVRDC)	Tainan, Taiwan		61,000 accessions from 155 countries, including about 12,000 accessions of indigenous vegetables.
DOA Genebank	Pathum Thani, Thailand	https://www.doa.go.th/genebankthailand/	
Banque Nationale de Gènes de Tunisie	Tunis, Tunisia	http://www.bng.nat.tn/	100 vegetable species of which 37 are cultivated in Tunisia.
Aegean Agricultural Research Institute, Biodiversity and Genetic Resources Department	Izmir, Turkey		Over 2400 Cucurbitaceae
National Center for Plant Genetic Resources of Ukraine	Kharkiv, Ukraine		74,400 specimens belonging to 342 crops and 739 botanical plant species.
UK Vegetable Genebank	University of Warwick, UK	https://warwick.ac.uk/fac/sci/lifesci/wcc/gru/genebank/collections/	14,000 samples of vegetable crops such as cauliflower, carrot, kale, lettuce and onions
TGRC, Tomato Genetics Resource Centre	University of California, Davis USA		Mainly wild tomato accessions collected first by C Rick. https://tgrc.ucdavis.edu/
USDA-ARS, Vegetable Germplasm Collection	USA	https://data.nal.usda.gov/dataset/national-plant-germplasm-system	Potato, carrot, onion, cucumber, cranberry and 3000 lettuce accessions (Pullman, WA site)

NPGRC, National Plant Genetic Resources Centre	Zambia		Over 2000 germplasm accessions of different crops including Maize, Sorghum, Millets, Rice, Finger millet, Pearl millet, Cowpea, Bean and Groundnut. Major root and tuber crops (cassava and sweet potato) and indigenous/local vegetable species have also been collected.

Note: A list of European genebanks can be found in Daunay *et al*, 2003 [5].

References

1. Ribeiro, C.; Reifschneider, F.; Carvalho, S.; Bianchetti, L.; Buso, G. Embrapa's Capsicum Breeding Program—Looking Back … into the Future. *Crop Breeding, Genetics and Genomics* **2019**, *2*, doi:10.20900/cbgg20200001.
2. Wang, H.; Li, X.; Song, J. Vegetable Genetic Resources in China. *Horticultural Plant Journal* **2018**, *4*, 83–88, doi:10.1016/j.hpj.2018.03.003.
3. Hammer, K.; Montesano, V.; Direnzo, P.; Laghetti, G. Conservation of Crop Genetic Resources in Italy with a Focus on Vegetables and a Case Study of a Neglected Race of Brassica Oleracea. *Agriculture* **2018**, *8*, 105, doi:10.3390/agriculture8070105.
4. Dzyubenko, N.I. Vavilov's Collection of Worldwide Crop Genetic Resources in the 21st Century. *Biopreserv Biobank* **2018**, *16*, 377–383, doi:10.1089/bio.2018.0045.
5. Daunay, M.-C.; Maggioni, L.; Lipman, E. *Solanaceae Genetic Resources in Europe: Report of Two Meetings, 21 September 2001, Nijmegen, The Netherlands ; 22 May 2003, Skierniewice, Poland; ECP/GR [European Cooperative Programme for Crop Genetic Resources Networks]*; IPGRI: Rom, 2003; ISBN 978-92-9043-579-2.
